# Patterns of intra-cluster correlation coefficients in school-based cluster randomised controlled trials of interventions for improving social-emotional functioning outcomes in pupils: a secondary data analysis of five UK-based studies

**DOI:** 10.1186/s12874-025-02574-6

**Published:** 2025-05-03

**Authors:** Kitty Parker, Michael Nunns, ZhiMin Xiao, Tamsin Ford, Paul Stallard, Willem Kuyken, Nick Axford, Obioha C. Ukoumunne

**Affiliations:** 1 NIHR Applied Research Collaboration South West Peninsula (PenARC), University of Exeter, Exeter, UK; 2 College of Medicine and Health, University of Exeter, Exeter, UK; 3 School of Health and Social Care, University of Essex, Colchester, UK; 4 Department of Psychiatry, University of Cambridge, Cambridge, UK; 5 Department for Health, University of Bath, Bath, UK; 6 Department of Psychiatry, University of Oxford, Oxford, UK; 7 NIHR Applied Research Collaboration South West Peninsula (PenARC), University of Plymouth, Plymouth, UK; 8 NIHR Applied Research Collaboration South West Peninsula (PenARC), University of Exeter, South Cloisters, St Luke’s Campus, Heavitree Rd, Exeter, EX1 2LU UK

**Keywords:** Cluster randomised trials, Intra-cluster correlation coefficient, Schools, Classrooms, Pupils, Mental health, Social-emotional functioning

## Abstract

**Background:**

The cluster randomised trial (CRT) design is increasingly used to evaluate the impact of school-based interventions for improving social-emotional functioning outcomes in pupils. Good knowledge is required on plausible values of the intra-cluster correlation coefficient (ICC) of the outcome to calculate the required sample size in such studies. Using data from five school-based CRTs in the UK, we estimate, and describe patterns in, ICCs for social-emotional functioning outcomes.

**Methods:**

Mixed effects linear regression models were fitted to estimate the ICC and variance components. Estimates for baseline data were obtained by fitting “null” models that had no predictor variables; estimates at follow-up were adjusted for trial arm status.

**Results:**

Five hundred and twenty-nine (529) ICCs were estimated. Variation across clusters in the outcomes was present at the school, year group and classroom levels. Overall, the ICCs were not markedly different between the primary and secondary school settings. Most of the school- and classroom-level ICCs were less than 0.04 for pupil-reported outcomes and less than 0.035 for parent-reported outcomes; a notable exception for pupil-reported outcomes was for outcomes that reflect a common experience shared by children, such as school climate, where the ICCs were as large as 0.1. The ICCs for teacher-reported outcomes (up to 0.1 at the school level and 0.2 at the classroom level) were larger than for pupil- and parent-reported outcomes. In the CRT that allocated schools to trial arms and only sampled one classroom from each school, the nominal school-level ICCs for teacher-reported outcomes took values up to 0.25. ICCs for teacher-reported measures of internalising behaviour problems and pro-social behaviour were larger than for externalising behaviour problems.

**Conclusions:**

When randomising school clusters, sub-sampling of lower-level clusters such as classrooms should be accounted for in the sample size calculation. Teacher-reported ICCs are likely to be greater than those for pupil- and parent-reported outcomes as teachers will often provide data for many or all pupils in a given school or classroom. Differences across reporter type and across outcomes need to be considered when specifying plausible values of the ICC to calculate sample size.

**Trial registration:**

STARS study (ISRCTN84130388); KiVa study (ISRCTN23999021); PACES study (ISRCTN23563048); PROMISE study (ISRCTN19083628); MYRIAD study (ISRCTN86619085).

**Supplementary Information:**

The online version contains supplementary material available at 10.1186/s12874-025-02574-6.

## Background

Social-emotional functioning represents the capacity to understand, experience, express, and manage emotions and to develop meaningful relationships with others [1]. It encompasses concepts including, but not restricted to, mental health, behaviour, well-being, emotional challenges, bullying, neurodiversity and self-esteem. As approximately half of adult mental disorders have their onset during adolescence [2], there has been an increased focus on improving social-emotional functioning in children and young people [1, 3, 4]. Schools are recognised for the role they can play in the promotion of health in children and young people [5–7]. They provide an ideal setting in which to intervene to support good social-emotional health during the key developmental years of a young person’s life and to evaluate such activities, given the time that children and adolescents spend in school.

The cluster randomised trial (CRT) design is increasingly used to evaluate the impact of interventions administered in the school setting for improving social-emotional functioning outcomes [8–15]. CRTs are studies in which entire clusters of participants such as health organisations, geographic areas or other organisations are allocated to trial arms and outcomes are measured on the individual participants within those clusters [16–20]. In school-based studies, the allocated clusters may be entire schools, year groups, classrooms or teachers [21]. In the school setting, the CRT design may be preferred to the traditional individual randomised trial (IRT) design in which individual participants are allocated because the interventions are naturally delivered to clusters of pupils (e.g., educational lessons, changes in school policy) or to avoid contamination between trial arms that might otherwise result if individual pupils are allocated within clusters [21].

An important consideration in CRTs is that the responses of participants in the same cluster tend to be more similar than those of participants from different clusters. The method used to calculate the total number of participants required in a CRT needs to take within-cluster similarity into account, because, as a result of this, each participant in a CRT contributes less information than each participant in a trial that randomises individual participants. The number of participants that would be required in an IRT needs to be multiplied by the design effect (*DE*) [17], also known as the variance inflation factor, to obtain the number of participants required in a CRT. For a simple design with continuous or binary outcomes, where the same number of participants (*n*) provide outcome data in each cluster, the design effect is:

$$DE=1+(n-1)\times \rho$$

where *ρ* is the intra-cluster correlation coefficient (ICC) of the outcome. The ICC quantifies the similarity or correlation between participant responses within clusters. It can be defined as the proportion of the total variation in the outcome that is between clusters as opposed to between individuals within clusters

$$\rho =\frac{{\sigma }_{b}^{2}}{{\sigma }_{b}^{2}+{\sigma }_{w}^{2}}$$

where ${\sigma }_{b}^{2}$ is the between-cluster variance component and ${\sigma }_{w}^{2}$ is the within-cluster variance component. The larger *ρ* is, the greater the design effect and, therefore, the greater the additional number of participants that need to be recruited in a CRT to allow for clustering.

In order to calculate the required sample size for a CRT, good knowledge is required on plausible values of *ρ* to assume for the outcome of interest and the type of cluster to be randomised [22]. The challenge of identifying good estimates of the ICC was highlighted in a systematic review of definitive school-based CRTs in the UK, where the assumed ICC for the primary outcome in the sample size calculation was often markedly different from the ICC estimated from the resulting study data [21]. This is consistent with findings in the wider CRT literature [23] and may reflect the lack of availability of relevant and precise estimates of the ICC at the time of sample size calculation. The rate of publication of school-based CRTs with health outcomes on pupils is increasing, but, despite there being a specific reporting item in the CONSORT statement for CRTs [24], many authors do not report estimates of *ρ* from their studies; just 55% (18/33) of papers published after the 2012 CONSORT-CRT extension were reported to have done so in the systematic review of definitive school-based CRTs [21].

Several papers have collated school-based ICCs for social-emotional functioning outcomes [25–29], mostly using data from studies undertaken in the United States. In a study reporting ICC estimates from school-based CRTs worldwide with health outcomes, the median school level ICC for social-emotional functioning outcomes (0.05) was not dissimilar to those for outcomes related to smoking, alcohol use, dental/oral health, infection disease, nutrition and violence, but was slightly higher than for physical activity (0.035), adiposity (0.027) and general health (0.025) outcomes [30]. The literature indicates that ICCs for social-emotional functioning outcomes range from around 0 to 0.1 [29–31], but this is too wide to usefully inform sample size calculations given that the design effect can be sensitive to the assumed ICC value, especially when large numbers of participants are sampled from each cluster. Greater knowledge is required on patterns in ICC values and the factors that are predictive of them.

Several considerations need to be made when specifying the ICC to calculate the sample size for school-based CRTs. First, when designing a CRT in the school setting, there are different levels at which cluster randomisation can be undertaken, including schools, year groups and classrooms. Randomising smaller, lower-level cluster types like classrooms has a greater risk of contamination between trial arms than if entire schools are randomised as, in the former scenario, pupils can interact between trial arms within the same school. Studies that randomise lower-level clusters are, however, potentially more efficient as they will typically include a larger number of allocated cluster units, given that there are, for example, more classroom units than school units [32]. The potential advantages and disadvantages of randomising at each level need to be considered when planning a school-based CRT. This requires knowledge of the components of variance and the ICC at the levels of clustering at which randomisation might be undertaken in the school setting, and an assessment of the potential risk of contamination resulting from allocating clusters at those levels [12, 33].

Second, sample size calculations are usually undertaken for CRTs that have a simple two-level data structure, explicitly recognising variation in the outcome at the level of randomisation (the allocated cluster) and the level of observation (the individual participant). In CRTs where school clusters (level 3 – highest level) are randomised, participation may be restricted to pupils (level 1 – lowest level) that are members of lower-level sub-clusters (level 2 – intermediate level), such as classrooms, that are sub-sampled to participate in the study [10, 11, 34–36]. When planning such studies, outcome variation at the randomisation level (school), the intermediate/sub-sampling level (e.g., classroom) and observation level (pupil) should be taken into account [37]. The design effect for a CRT that has a three-level structure is impacted by the relative sizes of the components of variance at each level and the number of intermediate-level clusters sampled from each school [37–39]. In studies that randomise schools, the more intermediate-level cluster units sampled from each school the more efficient the design. Design effect formulae that are appropriate for cluster trials where intermediate level clusters are sub-sampled [37] should be used, but this requires knowledge of the components of variation (or ICC) at all levels of the design.

Finally, a characteristic feature of school-based trials of interventions for improving social-emotional functioning of pupils is the reporting of outcomes by different sources, specifically by the pupils themselves, parents/carers and teachers. The components of variance and ICC may depend on who reports the outcome and this needs to be considered when specifying an appropriate ICC value for the sample size calculation for a planned school-based CRT.

In the review of ICC estimates from school-based CRTs worldwide, outcomes categorised under social-emotional functioning were the most common, with just over a fifth of the 246 studies reporting such outcomes as primary [30]. The review used characteristics of the studies (e.g., region, education stage, type of cluster allocated) to explore patterns in ICC values and identify aspects of the design and study setting that determine their values. The relationships examined between those characteristics and the ICC were potentially confounded by other design and contextual differences across the studies. Furthermore, the analysis only used data that were reported in the papers.

The current paper reports findings from a secondary analysis to estimate the intra-cluster correlation coefficient and components of variance for social-emotional functioning outcomes using raw data from five school-based CRTs in the UK. The use of raw datasets from CRTs provides more control over the level of detail that can be reported on the ICCs and the method of their calculation as the data for the analysis are not restricted to the information reported in the publications. For example, the components of variance at the school, year group, classroom and pupil levels, statistics that are generally not reported, are readily calculated. The analysis of raw data also facilitates the use of within-study information to examine characteristics that are potentially associated with the ICC. Finally, through use of raw data, there is the opportunity to comprehensively report the ICC for all relevant pupil outcomes reported by different people in those studies.

## Methods

### Datasets

The datasets used in this secondary analysis are from five published school-based CRTs undertaken in the UK that evaluated interventions for improving pupils’ social-emotional functioning outcomes [8, 11, 14, 15, 35]. Ethics approval and consent from participants were obtained by the investigators for the original studies. Permission to use these data in the current paper was granted by the principal investigator for each study, while individual participant information and consent permitting such future secondary analyses were covered by the original agreements. All cluster-level and individual-level data were anonymised in the original studies. Ethical approval for use of the datasets was granted by the University of Exeter Medical School Research Ethics Committee.

Table 1 summarises the characteristics of the CRTs, Table 2 summarises the demographic characteristics of participants and Supplementary Table S1 provides information regarding the outcomes, measures, reporters and the methods used to calculate total scores for the measures in each study. The datasets are described below, referring to studies by their acronym throughout.

**Table 1:** Characteristics of the school-based cluster randomised trials at randomisation

Author, year (Study acronym)	Education setting; *location*	Cluster unit allocated	Measurement time points (months)	Number of schools	Number of year groups	Number of classes	Number of pupils
Ford, 2019 [11] (STARS)	Primary schools; *South West England*	Schools (1 class sampled from each school)	0, 9, 18, 30	80	not applicable	80	2075
Axford, 2020 [8] (KiVa)	Primary schools; *Wales*	Schools	0, 12	22	not applicable	146	3214
Stallard, 2014 [15] (PACES)	Primary schools; *South West England* (*within 50-miles of the University of Bath*)	Schools	0, 6, 12	40	not applicable	73	1448
Stallard, 2012 [14] (PROMISE)	Secondary schools; *East Midlands and South West England*	Year groups	0, 6, 12	8	28	225	5761
Kuyken, 2022 [35] (MYRIAD)	Secondary schools; *England*, *Northern Ireland*, *Scotland*, *Wales*	Schools	0, 12, 19, 24	85	not applicable	346	8376

**Table 2:** Demographic characteristics of participants (*N*—sample size for variable)

STARS study						
Characteristic			*N*	Intervention	*N*	Control
Female, *n* (%)			1037	483 (46.6)	1038	491 (47.3)
Age in years, mean (SD)			1037	6.2 (1.4)	1038	6.4 (1.3)
White, *n* (%)			721	689 (95.6)	701	663 (94.6)
Eligible for free school meals (Yes), *n* (%)			595	70 (11.8)	502	64 (12.7)
KiVa study						
Characteristic			*N*	Intervention	*N*	Control
Female, *n* (%)			1428	717 (50.2)	1409	684 (48.5)
Age in years, mean (SD)			1423	8.8 (1.1)	1394	8.9 (1.2)
White, *n* (%)			1339	1176 (87.8)	1104	1018 (92.2)
Eligible for free school meals (Yes), *n* (%)			1353	237 (17.5)	1151	220 (19.1)
PACES study						
Characteristic	*N*	Health-led FRIENDS	*N*	School-led FRIENDS	*N*	Control
Female, *n* (%)	489	234 (47.9)	472	235 (49.8)	401	231 (57.6)
British White, *n* (%)	483	455 (94.2)	461	439 (95.2)	390	359 (92.1)
PROMISE study						
Characteristic	*N*	Classroom based CBT	*N*	Attention control	*N*	Control
Female, *n* (%)	1753	873 (50)	1673	824 (49)	1604	770 (48)
Age in years, mean (SD)	1753	14.1 (1.1)	1673	14.0 (1.0)	1604	13.9 (1.2)
White, *n* (%)	1582	1372 (86.7)	1521	1271 (83.6)	1480	1275 (86.1)
MYRIAD study						
Characteristic			*N*	Intervention	*N*	Control
Female, *n* (%)			4088	2350 (57.5)	3994	2159 (54.1)
Age in years, mean (SD)			4232	12.2 (0.6)	4144	12.2 (0.6)
White, *n* (%)			4145	3237 (78.1)	4048	2965 (73.2)

Percentages for categorical characteristics based on using the number of non-missing participants as the denominator

#### STARS

Supporting Teachers and childRen in Schools (*STARS*) [11] was undertaken in primary schools in the South West of England. The aim of the study was to evaluate whether the Incredible Years® Teacher Classroom Management (TCM) programme [40] improves children’s mental health, behaviour and enjoyment of school. Participants were pupils aged 4–9 years (Reception to Year 4). The study used a two-arm parallel CRT design that recruited three cohorts of schools (clusters), one for each of the 2012/13, 2013/14 and 2014/15 academic years. Schools were randomised to either the TCM programme (intervention arm) or teaching-as-usual (control arm) (Table 1). One classroom and its teacher were sub-sampled from each recruited school for participation. Eighty (80) schools were randomised, and 2075 pupils were recruited to the study: 40 schools (1037 pupils) in the intervention arm and 40 (1038 pupils) in the control arm. The TCM programme was delivered to teachers in the intervention arm in six whole-day sessions, spread over 6 months. Outcome data were collected on the pupils at baseline (0), 9, 18, and 30 months. Teacher-reported outcomes were provided by the same teacher for all pupils in a given classroom at the baseline and 9-month assessments. *Social-emotional functioning* was measured using the Strengths and Difficulties Questionnaire (SDQ) [41], providing a *total difficulties score* and subscale scores for *emotional symptoms*, *conduct problems*, *hyperactivity*, *peer problem* and *prosocial behaviour*. Parent- and teacher-reported versions of the SDQ were administered. *Pupil behaviour* was measured using the Pupil Behaviour Questionnaire (PBQ) [42], completed by the class teacher. *School climate* was measured using the pupil-reported ‘How I Feel About My School’ (HIFAMS) [43] questionnaire (Supplementary Table S1).

#### KiVa

*KiVa* [8] was undertaken in primary schools in Wales. The study evaluated the effectiveness of the 'Kiusaamista Vastaan' (*KiVa*) programme [44] to prevent and address bullying in schools. Participants were pupils aged 7–11 years (school Years 3 to 6). The study used a two-arm parallel CRT design with a waitlist (delayed intervention) control arm. Schools (clusters) were randomised to KiVa (intervention arm) or usual school provision (control arm) (Table 1). Schools were recruited in the middle of the 2012/13 academic year, with outcomes measured at the end of the 2013/14 academic year. Twenty-two (22) schools were randomised with 146 classrooms and 3214 pupils included in the study: 11 schools (77 classrooms, 1578 pupils) in the intervention arm and 11 schools (69 classrooms, 1636 pupils) in the control arm. Outcome data were collected at baseline (0 months) and 12 months. The outcomes were: *bullying victimisation* and *bullying perpetration* measured by the Olweus Bully/Victim Questionnaire (OBVQ) [45] and the KiVa student online survey [46], reported by the pupils; and *social-emotional functioning* measured using the Strengths and Difficulties Questionnaire (SDQ) [41], completed by the class teacher (Supplementary Table S1).

#### PACES

*PACES* [15] was undertaken in primary schools in the South West of England. The study evaluated the effectiveness of a classroom-based cognitive behavioural therapy (CBT) prevention programme (FRIENDS for life [47]) for reducing anxiety symptoms in children. Participants were pupils aged 9–10 years (school Year 5). The study used a three-arm parallel CRT design and took place between September 2011 and July 2012. Schools (clusters) were randomised to either receive school-led FRIENDS (led by teachers or school staff), health-led FRIENDS (led by trained health facilitators), or usual school provision (Table 1). Forty (40) schools were randomised, and 73 classrooms and 1448 pupils were included in the study: 14 schools (25 classrooms, 497 pupils) in the school-led FRIENDS arm; 14 schools (26 classrooms, pupils 509) in the health-led FRIENDS arm; and 12 schools (22 classrooms, pupils 442) in the control arm. Outcomes were measured at baseline (0), 6 and 12 months. *Symptoms of anxiety and low mood* were measured by the 30-item Revised Child Anxiety and Depression Scale (RCADS-30) [48], with a *total anxiety* score and subscale scores for *separation anxiety disorder*, *social phobia*, *generalised anxiety disorder*, *panic disorder*, *obsessive compulsive disorder*, and *low mood* (*major depressive disorder*). The RCADS-30 measure was reported separately by the pupil and the parent (RCADS-30-P). *Worry* was measured using the Penn State Worry Questionnaire for Children [49], reported by the pupil. *Self-worth and acceptance* was measured using the Rosenberg Self-Esteem Scale [50], reported by the pupil. *Bullying victimisation* was measured using the Olweus Bully/Victim Questionnaire (OBVQ) [45], reported by the pupil. *Life satisfaction* was measured using the Child Health Utility instrument (CHU9D) [51], reported by the pupil. *Social-emotional functioning* was measured by the Strengths and Difficulties Questionnaire (SDQ) [41], reported separately by the parent and the class teacher (Supplementary Table S1).

#### PROMISE

*PROMISE* [14] was undertaken in secondary schools in the East Midlands and South West of England. The study evaluated the effectiveness of classroom-based CBT (*The Resourceful Adolescent Programme* [52]) for reducing symptoms of depression, using an attention control arm for comparison (Personal, Social, and Health Education (PSHE) delivered by class teacher aided by two facilitators) and a usual school provision control arm. Participants were aged 12–16 years (school Years 8–11). The study used a three-arm parallel CRT design, allocating year groups (clusters) to either the CBT intervention, attention control, or usual school provision (Table 1). Twenty-eight (28) year groups from 8 schools with 225 classrooms and 5761 pupils were randomised: 10 year groups (79 classrooms, 2032 pupils) to CBT; 9 year-groups (73 classrooms, 1920 pupils) to attention control; and 9 year groups (73 classrooms, 1809 pupils) to usual school provision. Outcomes were measured at baseline (0), 6 and 12 months as follows: *symptoms of low mood* using the Short Mood and Feelings Questionnaire (SMFQ) [53], reported by the pupil; *negative thinking* using the *Personal Failure subscale* of the Children’s Automatic Thoughts Scale (CATS) [54], reported by the pupil; *self-worth and acceptance* using the Rosenberg Self-Esteem Scale [50], reported by the pupil; *anxiety* measured by the 30-item Revised Child Anxiety and Depression Scale (RCADS-30) [48], reported by the pupil; and *school connectedness* measured by the Psychological Sense of School Membership (PSSM) scale [55], reported by the pupil (Supplementary Table S1).

#### MYRIAD

MYRIAD [35] was a parallel arm CRT undertaken in secondary schools across the UK. The study evaluated the effectiveness of school-based mindfulness training (intervention arm) for improving student’s mental health, compared to teaching-as-usual (control arm). Participants were pupils aged 11–14 years (school Years 7–9). Schools (clusters) were randomised to the mindfulness training (intervention) arm or the control arm (Table 1). Classrooms within schools were selected to participate, sub-sampling a sufficient number of classrooms to recruit the required number of pupils in each school. Eighty-five (85) schools were randomised with 346 classrooms and 8376 pupils included in the study: 42 schools (169 classrooms and 4144 pupils) in the intervention arm, and 43 schools (177 classrooms and 4232 pupils) in the control arm. Baseline data were collected on the three pupil-reported co-primary outcomes (*risk for depression* using the Centre for Epidemiologic Studies for Depression Scale (CES-D) [56], *social-emotional functioning* using the Strengths and Difficulties Questionnaire (SDQ) [41], and *well-being* using the Warwick-Edinburgh Mental Well-being Scale (WEMWBS) [57]). These and other secondary outcomes were administered at 12, 19 and 24 months post-baseline. The secondary outcomes were: *executive function* measured by the Behaviour Rating Inventory of Executive Function (BRIEF-2) [58], reported separately by both the pupil and the class teacher; *anxiety* using 37 of the 47 items (excluding the 10 items on the Low Mood subscale) of the Revised Child Anxiety and Depression Scale (RCADS) [59], reported by the pupil; *self-harm and suicidal ideation* using measures devised for the study [35], reported by the pupil; *school climate subscales (school leadership and involvement, respectful climate, peer climate, caring adults)* from the School Climate and Connectedness Survey (SCCS) [60], reported by the pupil; *mindfulness skills* using the Child and Adolescent Mindfulness Measure (CAMM) [61], reported by the pupil (Supplementary Table S1).

### Data analysis

Data analysis was undertaken using Stata software [62]. Mixed effects (“multilevel”) linear regression models were fitted to continuous and binary outcomes to estimate the variance components and intra-cluster correlation coefficient (ICC). As a mixed model is used, the ICCs for binary outcomes are estimated on the proportions scale.

A 2-level mixed effects model was fitted to estimate the ICCs for the *STARS* study that had only a single level of clustering at the school level, since only one classroom was sampled from each school:

$${Y}_{il}=\alpha +{s}_{i}+{e}_{il}$$

where ${Y}_{il}$ is the outcome for the $l$ ^th^ individual in the $i$ ^th^ school (cluster); $\alpha$ is the constant; ${s}_{i}$ is the random effect of the $i$ ^th^ school, assumed to be Normally distributed with mean zero and constant variance ${\sigma }_{s}^{2}$; and ${e}_{il}$ is the residual effect of the *l*^th^ pupil in the $i$ ^th^ school assumed to be Normally distributed with mean zero and constant variance ${\sigma }_{e}^{2}$.

The school-level ICC (*ρ*_*s*_) was calculated from the between-cluster (${\sigma }_{s}^{2})$ and within-cluster (${\sigma }_{e}^{2}$) components of variances as:

$${\rho }_{s}=\frac{ {\sigma }_{s}^{2}}{{\sigma }_{s}^{2}+{\sigma }_{e}^{2}}$$

Three-level mixed effects models were fitted to estimate the ICCs for the *KiVa*, *PACES* and *MYRIAD* studies that had two levels of clustering (school and classroom):

$${Y}_{ikl}=\alpha +{s}_{i}+{c}_{ik}+ {e}_{ikl}$$

where ${Y}_{ikl}$ is the outcome for the $l$ ^th^ pupil in the $k$ ^th^ classroom, in the $i$ ^th^ school; $\alpha$ is the constant; ${s}_{i}$ is the random effect of the $i$ ^th^ school, assumed to be Normally distributed with mean zero and constant variance ${\sigma }_{s}^{2}$; ${c}_{ik}$ is the random effect of the $k$ ^th^ classroom in the $i$ ^th^ school, assumed to be Normally distributed with mean zero and constant variance ${\sigma }_{c}^{2}$; and ${e}_{ikl}$ is the residual effect of the *l*^th^ pupil in the $k$ ^th^ classroom, in the $i$ ^th^ school, assumed to be Normally distributed with mean zero and constant variance ${\sigma }_{e}^{2}$.

The school-level ICC (*ρ*_*s*_) was calculated from the variance components as:

$${\rho }_{s}=\frac{ {\sigma }_{s}^{2}}{{\sigma }_{s}^{2}+{\sigma }_{c}^{2}{ + \sigma }_{e}^{2}}$$

and the classroom-level ICC (*ρ*_*c*_) was calculated as:

$${\rho }_{c}=\frac{ {\sigma }_{c}^{2}}{{\sigma }_{c}^{2}{ + \sigma }_{e}^{2}}$$

This definition of the classroom-level ICC is appropriate to use when designing cluster randomised trials where allocation of classroom clusters is stratified by school membership.

Four-level mixed effects models were fitted to estimate the ICCs for the *PROMISE* study that had three levels of clustering (school, year group and classroom):

$${Y}_{ijkl}=\alpha +{s}_{i}+{{g}_{ij}+{c}_{ijk}+e}_{ijkl}$$

where ${Y}_{ijkl}$ is the outcome for the $l$ ^th^ pupil in the $k$ ^th^ classroom, in the $j$ ^th^ year group, in the $i$ ^th^ school; $\alpha$ is the constant; ${s}_{i}$ is the random effect of the $i$ ^th^ school, assumed to be Normally distributed with mean zero and constant variance ${\sigma }_{s}^{2}$; ${g}_{ij}$ is the random effect of the $j$ ^th^ year group in the $i$ ^th^ school, assumed to be Normally distributed with mean zero and constant variance ${\sigma }_{g}^{2}$; ${c}_{ijk}$ is the random effect of the $k$ ^th^ classroom, in the $j$ ^th^ year group in the $i$ ^th^ school, assumed to be Normally distributed with mean zero and constant variance ${\sigma }_{c}^{2}$; and ${e}_{ijkl}$ is the residual effect of the *l*^th^ pupil in the $k$ ^th^ class, in the $j$ ^th^ year group in the $i$ ^th^ school, assumed to be Normally distributed with mean zero and constant variance ${\sigma }_{e}^{2}$.

The school-level ICC (*ρ*_*s*_) was calculated from the variance components as:

$${\rho }_{s}=\frac{ {\sigma }_{s}^{2}}{{\sigma }_{s}^{2}+{\sigma }_{g}^{2}+{\sigma }_{c}^{2}+{\sigma }_{e}^{2}}$$

the year group-level ICC (*ρ*_*g*_) was calculated as:

$${\rho }_{g}=\frac{ {\sigma }_{g}^{2}}{{\sigma }_{g}^{2}+{\sigma }_{c}^{2}+{\sigma }_{e}^{2}}$$

and the classroom-level ICC was calculated as:

$${\rho }_{c}=\frac{ {\sigma }_{c}^{2}}{{\sigma }_{c}^{2}+{\sigma }_{e}^{2}}$$

The definitions of the year group-level and classroom-level ICCs are appropriate to use when designing cluster randomised trials where allocation of clusters at those levels is stratified by higher level clusters.

ICC estimates at the baseline time point were obtained by fitting “null” or “empty” models that had no predictor variables. ICC estimates at follow-up were adjusted for trial arm status by adding the variable as a predictor (fixed effect) to the above models. We did not adjust for other covariates since adjustment factors will differ across trials. The ICC estimates reported provide a common base of information that will be useful for planning future studies.

## Results

**Fig. 1:**
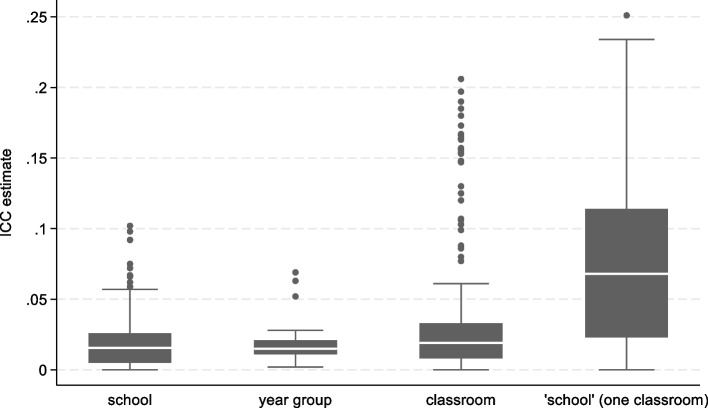
Distribution of ICCs by type of cluster

Altogether, 529 ICCs were estimated across the 5 studies: 222 at the school level; 30 at the year group level; 221 at the classroom level; and 56 at the “school level”, estimated from the *STARS* study that randomised schools and included only one classroom from each school. The box plot in Fig. 1 summarises the distribution of ICCs by cluster level. The median ICC was similar at the school (0.0155) and year group levels (0.015), and slightly higher at the classroom level (0.019). The median “school-level” ICC for the STARS study was much larger (0.068); the possible reasons for this are discussed later in this section.

**Fig. 2:**
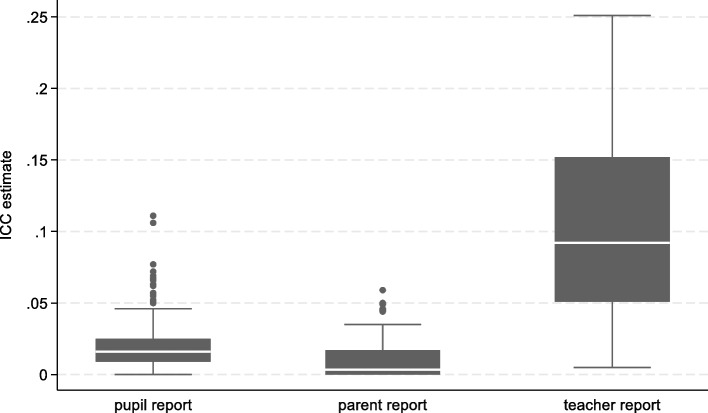
Distribution of ICCs by reporter type

ICCs were estimated for 333 pupil-reported outcomes, 102 parent-reported outcomes and 94 teacher-reported outcomes. The box plot in Fig. 2 summarises the distribution of ICCs by reporter type. Most ICCs were less than 0.04 for pupil-reported outcomes (median (90% range): 0.016 (0 to 0.05)) and less than 0.035 for parent-reported outcomes (median (90% range): 0.0035 (0 to 0.045)). Teacher-reported ICCs were as large as 0.25 (median (90% range): 0.092 (0.023 to 0.202)).

The median ICC was similar between the primary (*N* = 244) and secondary school (*N* = 285) settings (0.017 and 0.018, respectively).

The remainder of this section describes the results separately for each study to highlight the nuances of patterns in the ICC values.

### STARS study

The estimated school-level ICC and the variance components at the “school” and pupil levels from the STARS study are reported in Supplementary Table S2 for the Strengths and Difficulties Questionnaire (SDQ) (teacher- and parent-report) and the teacher-reported Pupil Behaviour Questionnaire, and in Supplementary Table S3 for the pupil-reported “How I Feel About My School (HIFAMS)” measure. The ICCs should be interpreted in the context that only one classroom was sampled from each randomised school, and it is not possible to separate the variation at the classroom level from that at the school level. The ICC, therefore, reflects variation at both the school and classroom levels combined. The “school-level” ICCs ranged from 0 to 0.251. The ICCs for the teacher-reported SDQ total score and SDQ subscales (range 0.053 to 0.251) were markedly higher than those for the corresponding parent-reported measures (range 0 to 0.049). This might be expected given that, unlike the parents, who only report for their own child, each teacher completed the SDQ for all pupils in their school (at least at baseline and 9 months follow-up) and the higher ICCs for the teacher report may reflect variability in their tendency to give lower or higher ratings generally. The ICCs for the teacher-reported SDQ subscales were notably lower for externalising behaviour (Conduct Problems and Hyperactivity) (median = 0.085) than for internalising behaviour (Emotional and Peer Problems) (median = 0.134) and prosocial behaviour (median = 0.213). The externalising behaviours assessed by the SDQ are more overt and easily recognised compared to the internalising behaviours. This may result in greater variability across schools in the teachers’ ability to detect pupils’ internalising behaviour problems. In keeping with the conceptual overlap between the measures, the ICCs for the teacher-reported Pupil Behaviour Questionnaire (median = 0.078) were notably similar in size to those for the SDQ Conduct and Hyperactivity subscales scores. The ICCs for the pupil-reported “How I Feel About My School” measure, ranged from 0.052 to 0.111. The fact that the concept potentially reflects a common shared experience of the school may account for these ICCs being higher than for the parent-reported outcomes.

### KiVa study

The school-level and classroom-level ICCs and components of variance for the *KiVA* study are reported in Supplementary Table S4 for the teacher-reported SDQ subscales and pupil-reported bullying-related outcomes. The school-level ICCs ranged from 0.005 to 0.092 and the classroom-level ICCs from < 0.001 to 0.206; the larger values at the classroom level are consistent with the notion that the ICC is generally larger for clusters that are naturally smaller in size [63]. Unlike STARS which was also undertaken in primary schools, in KiVa, more than one classroom was sampled from each school, and it was possible to separately estimate components of variance at the school and classroom levels. This explains why the school-level ICCs for the teacher-reported SDQ subscales in KiVa are markedly smaller than the nominal ones reported for the STARS study.

### PACES study

School- and classroom-level ICCs and components of variance for the PACES study are reported for the parent- and pupil-reported Revised Child Anxiety and Depression Scale (RCADS-30) (in Supplementary Table S5), the parent-reported Strengths and Difficulties Questionnaire (SDQ) (in Supplementary Table S6) and the pupil reports of bullying victimisation, worry, self-esteem and life satisfaction (in Supplementary Table S7). The school-level ICCs were often very low with several zero values. The classroom-level ICCs for the pupil-reported RCADS-30 (median = 0.029) were notably larger than those for the corresponding parent report (median = 0), many of which were zero.

### PROMISE study

School-, year group- and classroom-level ICCs and components of variance from the PROMISE study are presented for pupil-reported outcomes in Supplementary Table S8. The school-level ICCs ranged from 0 to 0.016 (median = 0.005), the year group-level ICCs from 0.002 to 0.069 (median = 0.015) and the classroom-level ICCs from 0.011 to 0.035 (median = 0.0215). The pattern of clusters with smaller natural size having larger ICC values is generally observed here. The school- and classroom-level ICCs for the pupil-reported 30-item Revised Child Anxiety and Depression Scale (RCADS-30) in this secondary school setting (medians 0.005 and 0.022, respectively) were not dissimilar in size to those of the same measure in the primary school-based PACES study (medians 0.002 and 0.029, respectively).

### MYRIAD study

School- and classroom-level ICCs and components of variance from the MYRIAD study are reported in Supplementary Tables S9 to S13. The ICCs for the teacher-reported Strengths and Difficulties Questionnaire were markedly larger than those for the corresponding pupil reports, particularly at the classroom level where they ranged from 0.077 to 0.197 for teacher reports and from < 0.001 to 0.021 for pupil reports (Supplementary Table S9). The same finding of larger ICCs for teacher-reported outcomes was observed for the Behaviour Rating Inventory of Executive Function (Supplementary Table S10). These findings are expected given that in the MYRIAD study teachers reported outcomes for multiple pupils in the same school and often the same classroom.

The school-level ICCs for the pupil-reported Revised Child Anxiety and Depression Scale were larger in MYRIAD (median (range) = 0.0245 (0.016 to 0.040) – see Supplementary Table S11) than the corresponding ICCs in the PROMISE study (median (range) = 0.005 (0 to 0.014) – see Supplementary Table S8), which was also conducted in secondary schools. The opposite finding occurred for classroom-level ICCs with smaller values in MYRIAD (median (range) = 0.006 (0 to 0.020)) than in PROMISE (median (range) = 0.022 (0.012 to 0.035)). A further notable result of the MYRIAD study is that while the ICCs for teacher-reported outcomes were clearly larger at the classroom level than at the school level, those for the pupil-reported outcomes were generally slightly larger at the school-level than at the classroom level, in contrast to what is generally expected when comparing ICCs between larger and smaller cluster types. The between-cluster component of variance was generally larger at the school level than at the classroom level for pupil-reported outcomes. Taken together, these *MYRIAD* study findings indicate that school-level factors may be stronger determinants of pupil outcomes than classroom-level factors in secondary schools. In primary schools, children stay with the same classroom all day, whereas in secondary schools the structure is more fluid, with children moving from class to class and from teacher to teacher. Consequently, variation at the classroom level may have less salience for secondary schools than it has for primary schools, and school-level variation may assume greater importance relative to classroom-level variation in secondary schools. A further notable contextual factor is that, unlike the other four studies, the *MYRIAD* study recruited schools from across the entire UK. This could have resulted in a wider range of school characteristics which may explain the increased importance of school-level variation for *MYRIAD*.

The ICCs for the pupil-reported school climate outcomes (Supplementary Table S13) (school-level ICCs ranged from 0.032 to 0.072) were notably larger than the ICCs for other pupil-reported outcomes. As found in the *STARS* study for the “How I Feel About My School” measure, the larger ICCs for school climate in *MYRIAD* may reflect the fact that the responses capture the shared experience of pupils in the same school or classroom.

## Discussion

Using raw data from five school-based cluster randomised controlled trials we have reported estimates of the variance components and intra-cluster correlation coefficients at different levels of clustering for social-emotional functioning outcomes in children. The estimates indicate that notable variation in outcomes is present at the school, year group and classroom levels. When calculating the sample size for planned CRTs, the impact of the size of the variance components at different levels should be accounted for [37].

The ICCs for clustering at the classroom level tended to be larger than at the school level; this is consistent with previous findings that the ICC is generally larger for clusters that are naturally smaller in size [63, 64]. The exception to this was the MYRIAD trial where the vast majority of school-level ICCs for pupil-reported mental health outcomes (SDQ and RCADS) were larger than the corresponding classroom-level ICCs. Unlike the other four CRTs that only included schools from one or two regions, the MYRIAD trial recruited a large number of schools from across the entire UK. This suggests that larger ICCs might be expected in studies that span a wider geographic area and recruit diverse schools.

Comparison between the *STARS* and *KiVa* studies highlighted the impact that the design can have on the ICC. In both studies, primary schools were the units of randomisation, but whereas in *STARS* only one classroom was included from each school, in *KiVa* multiple classes were included. The nominal school-level ICCs for the teacher-reported SDQ in *STARS* were markedly larger than the school-level ICCs in *KiVa*. Large teacher-reported school-level ICCs for social-emotional functioning outcomes on pupils were reported in another school-based CRT that sampled one classroom per school [10]. In studies that sample only one classroom per school, the variation at school level cannot be separated from the variation at the classroom level. When using estimates of school-level ICCs from previous studies to plan new trials, investigators should establish whether, and how many, classrooms were sub-sampled from each school as this will have an impact on the relevance of the ICCs for the planned trial.

School-based trials with social-emotional functioning outcomes on pupils are characterised by the collection of data from different responders, including the pupils themselves, their parents and their teachers. In such studies, the teachers are often responsible for reporting outcomes for all pupils in a given classroom or school cluster; consequently, the ICC may be markedly larger for teacher-reported outcomes than pupil- and parent-reported outcomes. Differences across reporters in the ICC estimates for the same outcome may also be partly due to their having different perspectives on how they subjectively rate a particular outcome [65]. These aspects need to be considered when specifying plausible values of the ICC for sample size calculation for a planned CRT.

Along with the type of cluster, the outcome is a direct determinant of the size of the ICC. Analyses of the teacher report of the Strengths and Difficulties Questionnaire (SDQ) from the *STARS* and *KiVa* studies indicated that the ICCs differ within the same measure across subscales that quantify different outcomes. At the same time, similarity between the ICCs for the teacher-reported Conduct Problems and Hyperactivity subscales on the SDQ and the ICC for the teacher-reported Pupil Behaviour Questionnaire measure in the *STARS* study suggests that ICCs may be similar between different measures of the same or similar outcomes.

For studies that included the teacher-reported Strengths and Difficulties Questionnaire, ICCs were generally largest for the *prosocial behaviour* subscale. Dong and colleagues found that school-level ICCs for teacher-reported prosocial behaviour measured by the Teacher Observation of Classroom Adaption—Checklist (TOCA-C) ranged from 0.29 to 0.54, while all other teacher-reported social-emotional functioning outcomes had lower school-level ICCs ranging from 0.03 to 0.23 [25]. These findings may reflect greater variation across teachers in their ability to recognise prosocial behaviour, and the fact that many of its aspects (e.g., helping, sharing, consoling and comforting) are exhibited more frequently outside of the classroom/learning environment where the teacher may not observe them [66]. Reported behaviours that are more difficult to observe and measure may be more susceptible to variation across teachers resulting in larger ICC estimates for these outcomes [66]. The lower ICC for teacher report of pupil conduct on the SDQ may be due to there being less variation across teachers regarding their awareness of *conduct* and *hyperactivity* problems than there is for their awareness of *emotional difficulties*, *peer problems* and *prosocial behaviour* [67]. All teachers may be aware and concerned with challenging behaviours in schools and rate the presence of these behaviours more similarly than other behaviours. Furthermore, schools have behavioural policies which may provide more guidance on how to handle conduct problems, encouraging more consistency across teachers in this regard compared with internalising behaviour problems [68].

Using different instruments, *STARS*, *PROMISE* and *MYRIAD* all measured pupil-reported school climate and connectedness. Particularly in *MYRIAD*, ICC estimates for school climate outcomes were markedly larger than those for pupil-reported mental health outcomes. Bradshaw and colleagues in a US study also noted high school-level ICCs for school climate outcomes, ranging from 0.04 to 0.1 [69]. Compared to mental health outcomes, school climate and school connectedness might be considered to be more directly impacted by the school environment [70, 71], which may explain why the ICCs for such outcomes are larger than mental health outcomes.

The findings in this secondary analysis indicate ballpark ranges that may be useful for testing the sensitivity of the required sample size in school-based CRTs to different assumed values of the ICC for socio-emotional functioning outcomes. For studies where outcomes are reported by pupils and parents, 0.04 and 0.035, respectively, are practical upper limits on plausible values for school-level and classroom-level ICCs. A notable exception for pupil-reported outcomes is for measures of school climate or school experience, where the ICCs can be as large as 0.1. The ICC for teacher-reported outcomes can be as large as 0.1 at the school level and as large as 0.2 at the classroom level, based on the same teacher rating many or all children within a given school or classroom. In CRTs that allocate schools to trial arms and only sample one classroom from each school, nominal school-level ICCs for teacher-reported outcomes may be as large as 0.25.

### Strengths and limitations

There are several strengths to this secondary data analysis. The ICCs were estimated using data from completed CRTs and, therefore, should be applicable to future studies as the schools and participants are more likely to be representative of those that take part in trials than other sources of ICCs [18] (p. 177). The reporting of components of variance at different levels of clustering helps facilitate the design of CRTs with more complex design structures. The included studies were undertaken recently enough for the ICC estimates to be relevant, and a range of social-emotional functioning outcomes were analysed. Furthermore, the settings of the studies span different UK regions and both primary and secondary school settings. Four of the five studies, however, drew their samples from only one or two regions, which may have resulted in underestimates of the school-level ICCs in the UK as a whole. Alongside this, they may provide more relevant ICC estimates for planning trials that are to be undertaken in a single region or area of the country, given the greater variability across schools in the UK-wide sample recruited to the MYRIAD study.

The focus of the study on the UK is a strength of this work, resulting in focussed and rich data in a specific setting. Simultaneously, this potentially limits the applicability of the findings internationally, especially to countries with different education systems. However, a recent review of ICCs in school-based trials revealed little evidence of marked differences in the size of the ICCs across world regions [30]. Despite being focussed on the UK, the findings of this secondary analysis will still be of global interest. The range of ICCs in the current study largely overlapped with the range of ICCs (0 to 0.217) collated for social-emotional functioning outcomes in that review [30]. Furthermore, other high and upper/middle income countries such as Australia have a similar school system to the UK, increasing the likelihood that the findings have applicability to those settings.

A further limitation is that it was only possible to include the five studies for which access to the data were immediately available for this secondary analysis; specifically, studies on which the authors were co-investigators or had worked. In the systematic review of definitive school-based CRTs in the UK with health outcomes, 11 studies other than those analysed in the current paper had social-emotional functioning outcomes [21]. Collectively, the five studies analysed in the current paper included schools from all four constituent countries of the UK. Two of the analysed studies (KiVA and MYRIAD) included schools from Wales and one (MYRIAD) included schools from Scotland; none of the other studies found in the systematic review included schools from those countries. All the analysed studies in the current paper included state schools and one (MYRIAD) included independent schools; this reflects the characteristics of the other studies included in the systematic review. Both males and females were included in the analysed studies in the current paper, reflecting the composition of other school-based CRTs with social-emotional functioning outcomes in the UK. The percentage in the White ethnic group category in the analysed studies was high (ranging from 75 to 95%) compared to the other studies with social-emotional functioning outcomes (ranging from 24 to 93%). Knowledge from other school-based CRTs with social-emotional functioning outcomes would further enrich knowledge of patterns in the ICC. Analyses of datasets that have diverse samples could investigate the extent to which the ICC differs across groups defined by ethnicity and gender. Some outcomes were only assessed in one dataset in the current study; analysis of these same outcomes using other datasets would help to establish the consistency of the ICC estimates. Outcomes of relevance to social-emotional functioning were not included in the datasets, such as body image [72]. Future research should expand and replicate this work with other relevant studies and examine patterns for outcomes that were not included here.

## Conclusions

Knowledge of the components of variance and intra-cluster correlation coefficient at different levels of clustering is required for calculating the target sample size for school-based cluster randomised controlled trials of interventions for improving social-emotional functioning outcomes in pupils. The estimates reported in this paper will greatly aid the design of such studies and contribute more generally to the knowledge of factors that impact on the size of the ICC. It is important that when planning studies, investigators consider: the type of cluster that is allocated; whether lower-level clusters are sub-sampled within the allocated cluster and the number that are sub-sampled; whether the outcome is reported by pupils, teachers or parents/carers; the concept that is quantified by the outcome measure; and whether the outcome relates to or reflects a common experience shared by all pupils in the same school or classroom.

## Supplementary Information

Supplementary Material 1.

## Data Availability

The datasets generated and/or analysed during the current study are not publicly available but are available from the authors on reasonable request.

## Abbreviations

BRIEF- 2: Behaviour rating inventory of executive function
CAMM: Child and adolescent mindfulness measure
CATS: Children’s automatic thoughts scale
CBT: Cognitive behaviour therapy
CES-D: Centre for epidemiologic studies for depression scale
CHU9D: Child health utility
CONSORT: Consolidated standards of reporting trials
CRT: Cluster randomised trial
DE: Design effect
HIFAMS: How I feel about my school
ICC: Intra-cluster correlation coefficient
IRT: Individual randomised trial
OBVQ: Olweus bully/victim questionnaire
PBQ: Pupil behaviour questionnaire
PSHE: Personal, social, and health education
PSSM: Psychological sense of school membership
RCADS: Revised child anxiety and depression scale
RCADS- 30: 30 Item revised child anxiety and depression scale
SCCS: School climate and connectedness survey
SDQ: Strengths and difficulties questionnaire
SMFQ: Short mood and feelings questionnaire
TCM: Teacher classroom management
TOCA-C: Teacher observation of classroom adaption – checklist
UK: United Kingdom
US: United States
WEMWBS: Warwick-edinburgh mental well-being scale

## References

[1] Cohen J, Onunaku N, Clothier S, Poppe J. Helping young children succeed: Strategies to promote early childhood social and emotional development. Denver: National Conference of State Legislatures; 2005.

[2] Kessler RC, Amminger GP, Aguilar-Gaxiola S, Alonso J, Lee S, Ustün TB. Age of onset of mental disorders: a review of recent literature. Curr Opin Psychiatry. 2007;20:359–64. 17551351 10.1097/YCO.0b013e32816ebc8c PMC1925038

[3] Banerjee R, McLaughlin C, Cotney J, Roberts L, Peereboom C. Promoting emotional health, well-being and resilience in primary schools. Wales: Public Policy Institute of Wales; 2016.

[4] Reinke WM, Stormont M, Herman KC, Puri R, Goel N. Supporting children’s mental health in schools: Teacher perceptions of needs, roles, and barriers. Sch Psychol Q. 2011;26:1–13.

[5] Bonell C, Humphrey N, Fletcher A, Moore L, Anderson R, Campbell R. Why schools should promote students’ health and wellbeing. BMJ. 2014;348:g3078. 25134103 10.1136/bmj.g3078

[6] Goesling B. A practical guide to cluster randomized trials in school health research. J Sch Health. 2019;89:916–25. 31506951 10.1111/josh.12826

[7] Langford R, Bonell CP, Jones HE, Pouliou T, Murphy SM, Waters E, et al. The WHO Health Promoting School framework for improving the health and well-being of students and their academic achievement. Cochrane Database Syst Rev. 2014;2014:CD008958. 24737131 10.1002/14651858.CD008958.pub2 PMC11214127

[8] Axford N, Bjornstad G, Clarkson S, Ukoumunne OC, Wrigley Z, Matthews J, et al. The effectiveness of the KiVa bullying prevention program in Wales, UK: results from a pragmatic cluster randomized controlled trial. Prev Sci. 2020;21:615–26. 32240480 10.1007/s11121-020-01103-9 PMC7305088

[9] Bonell C, Allen E, Warren E, McGowan J, Bevilacqua L, Jamal F, et al. Effects of the Learning Together intervention on bullying and aggression in English secondary schools (INCLUSIVE): a cluster randomised controlled trial. Lancet. 2018;392:2452–64. 30473366 10.1016/S0140-6736(18)31782-3 PMC6286420

[10] Connolly P, Miller S, Kee F, Sloan S, Gildea A, McIntosh E, et al. A cluster randomised controlled trial and evaluation and cost-effectiveness analysis of the Roots of Empathy schools-based programme for improving social and emotional well-being outcomes among 8-to 9-year-olds in Northern Ireland. Public Health Research. 2018;6(4). 29543419

[11] Ford T, Hayes R, Byford S, Edwards V, Fletcher M, Logan S, et al. The effectiveness and cost-effectiveness of the Incredible Years Teacher Classroom Management programme in primary school children: results of the STARS cluster randomised controlled trial. Psychol Med. 2019;49:828–42. 30017006 10.1017/S0033291718001484 PMC6425365

[12] Humphrey N, Barlow A, Wigelsworth M, Lendrum A, Pert K, Joyce C, et al. A cluster randomized controlled trial of the Promoting Alternative Thinking Strategies (PATHS) curriculum. J Sch Psychol. 2016;58:73–89. 27586071 10.1016/j.jsp.2016.07.002 PMC5019026

[13] Kidger J, Turner N, Hollingworth W, Evans R, Bell S, Brockman R, et al. An intervention to improve teacher well-being support and training to support students in UK high schools (the WISE study): A cluster randomised controlled trial. PLoS Med. 2021;18:e1003847. 34762673 10.1371/journal.pmed.1003847 PMC8629387

[14] Stallard P, Sayal K, Phillips R, Taylor JA, Spears M, Anderson R, et al. Classroom based cognitive behavioural therapy in reducing symptoms of depression in high risk adolescents: pragmatic cluster randomised controlled trial. BMJ. 2012;345:e6058. 23043090 10.1136/bmj.e6058 PMC3465253

[15] Stallard P, Skryabina E, Taylor G, Phillips R, Daniels H, Anderson R, et al. Classroom-based cognitive behaviour therapy (FRIENDS): a cluster randomised controlled trial to Prevent Anxiety in Children through Education in Schools (PACES). Lancet Psychiatry. 2014;1:185–92. 26360730 10.1016/S2215-0366(14)70244-5

[16] Campbell MJ, Walters S. How to Design, Analyse and Report Cluster Randomised Trials in Medicine and Health Related Research. Chichester: John Wiley and Sons; 2014.

[17] Donner A, Klar N. Design and Analysis of Cluster Randomization Trials in Health Research. Chichester: Wiley; 2000.

[18] Eldridge SM, Kerry S. A Practical Guide to Cluster Randomised Trials in Health Services Research. Chichester: John Wiley & Sons; 2012.

[19] Hayes R, Moulton L. Cluster Randomised Trials. Florida: CRC Press; 2009.

[20] Murray DM. Design and Anaylsis of Group-Randomized Trials. New York: Oxford University Press; 1998.

[21] Parker K, Nunns M, Xiao Z, Ford T, Ukoumunne OC. Characteristics and practices of school-based cluster randomised controlled trials for improving health outcomes in pupils in the UK: a methodological systematic review. BMC Med Res Methodol. 2021;21:152. 34311695 10.1186/s12874-021-01348-0 PMC8311976

[22] Eldridge SM, Ukoumunne OC, Carlin JB. The intra-cluster correlation coefficient in cluster randomised trials: a review of definitions. Int Stat Rev. 2009;77:378–94.

[23] Rutterford C, Taljaard M, Dixon S, Copas A, Eldridge S. Reporting and methodological quality of sample size calculations in cluster randomized trials could be improved: a review. J Clin Epidemiol. 2015;68:716–23. 25523375 10.1016/j.jclinepi.2014.10.006

[24] Campbell MK, Piaggio G, Elbourne DR, Altman DG. CONSORT 2010 statement: extension to cluster randomised trials. BMJ. 2012;345: e5661. 22951546 10.1136/bmj.e5661

[25] Dong N, Reinke WM, Herman KC, Bradshaw CP, Murray DW. Meaningful effect sizes, intraclass correlations, and proportions of variance explained by covariates for planning two- and three-level cluster randomized trials of social and behavioral outcomes. Eval Rev. 2016;40:334–77. 27694127 10.1177/0193841X16671283

[26] Hale DR, Patalay P, Fitzgerald-Yau N, Hargreaves DS, Bond L, Görzig A, et al. School-level variation in health outcomes in adolescence: analysis of three longitudinal studies in England. Prev Sci. 2014;15:600–10. 23793374 10.1007/s11121-013-0414-6

[27] Hedberg EC. Academic and behavioral design parameters for cluster randomized trials in kindergarten: an analysis of the Early Childhood Longitudinal Study 2011 Kindergarten Cohort (ECLS-K 2011). Eval Rev. 2016;40:279–313. 27354389 10.1177/0193841X16655657

[28] Sellström E, Bremberg S. Is there a “school effect” on pupil outcomes? A review of multilevel studies. Journal of epidemiology and community health. J Epidemiol Comm Health. 2006;60:149–55. 10.1136/jech.2005.036707 PMC2566146 16415266

[29] Shackleton N, Hale D, Bonell C, Viner RM. Intraclass correlation values for adolescent health outcomes in secondary schools in 21 European countries. SSM - Popul Health. 2016;2:217–25. 10.1016/j.ssmph.2016.03.005 PMC5757888 29349141

[30] Parker K, Nunns M, Xiao Z, Ford T, Ukoumunne OC. Intracluster correlation coefficients from school-based cluster randomized trials of interventions for improving health outcomes in pupils. J Clin Epidemiol. 2023;158:18–26. 36997102 10.1016/j.jclinepi.2023.03.020

[31] Bonell C, Jamal F, Harden A, Wells H, Parry W, Fletcher A. Systematic review of the effects of schools and school environment interventions on health: evidence mapping and synthesis. Public Health Research. 2013;1(1). 25642578

[32] Hemming K, Eldridge S, Forbes G, Weijer C, Taljaard M. How to design efficient cluster randomised trials. BMJ. 2017;358: j3064. 28710062 10.1136/bmj.j3064 PMC5508848

[33] James J, Thomas P, Cavan D, Kerr D. Preventing childhood obesity by reducing consumption of carbonated drinks: cluster randomised controlled trial. BMJ. 2004;328:1237. 15107313 10.1136/bmj.38077.458438.EE PMC416601

[34] Giles M, McClenahan C, Armour C, Millar S, Rae G, Mallett J, et al. Evaluation of a theory of planned behaviour–based breastfeeding intervention in Northern Irish schools using a randomized cluster design. Br J Health Psychol. 2014;19:16–35. 23350961 10.1111/bjhp.12024

[35] Kuyken W, Ball S, Crane C, Ganguli P, Jones B, Montero-Marin J, et al. Effectiveness and cost-effectiveness of universal school-based mindfulness training compared with normal school provision in reducing risk of mental health problems and promoting well-being in adolescence: the MYRIAD cluster randomised controlled trial. Evid Based Ment Health. 2022;25:99–109. 35820992 10.1136/ebmental-2021-300396 PMC9340028

[36] Norris E, Dunsmuir S, Duke-Williams O, Stamatakis E, Shelton N. Physically active lessons improve lesson activity and on-task behavior: A cluster-randomized controlled trial of the “Virtual Traveller” Intervention. Health Educ Behav. 2018;45:945–56. 29562763 10.1177/1090198118762106

[37] Rutterford C, Copas A, Eldridge S. Methods for sample size determination in cluster randomized trials. Int J Epidemiol. 2015;44:1051–67. 26174515 10.1093/ije/dyv113 PMC4521133

[38] Fazzari MJ, Kim MY, Heo M. Sample size determination for three-level randomized clinical trials with randomization at the first or second level. J Biopharm Stat. 2014;24:579–99. 24697506 10.1080/10543406.2014.888436

[39] Teerenstra S, Moerbeek M, van Achterberg T, Pelzer BJ, Borm GF. Sample size calculations for 3-level cluster randomized trials. Clin Trials. 2008;5:486–95. 18827041 10.1177/1740774508096476

[40] Webster-Stratton C, Reid MJ. The Incredible Years parents, teachers, and children training series: A multifaceted treatment approach for young children with conduct problems. In: Weisz JR, Kazdin AE, editors. Evidence-based psychotherapies for children and adolescents. 3rd ed. New York: Guilford Press; 2018. p. 122–41.

[41] Goodman R. Psychometric properties of the strengths and difficulties questionnaire. J Am Acad Child Adolesc Psychiatry. 2001;40:1337–45. 11699809 10.1097/00004583-200111000-00015

[42] Allwood M, Allen K, Price A, Hayes R, Edwards V, Ball S, et al. The reliability and validity of the pupil behaviour questionnaire: a child classroom behaviour assessment tool. Emot Behav Diffic. 2018;23:361–71.

[43] Allen K, Marlow R, Parker C, Rodgers L, Ukoumunne OC, Chan Seem E, et al. ‘How I Feel About My School’: the construction and validation of a method of wellbeing at school for primary school children. Clin Child Psychol Psychiatry. 2018;23:25–41. 28135832 10.1177/1359104516687612

[44] Salmivalli C, Kärnä A, Poskiparta E. Counteracting bullying in Finland: The KiVa program and its effects on different forms of being bullied. Int J Behav Dev. 2011;35:405–11.

[45] Olweus D. The Revised Olweus Bully / Victim Questionnaire. Bergen: University of Bergen; 1996.

[46] Kärnä A, Voeten M, Little TD, Poskiparta E, Alanen E, Salmivalli C. Going to scale: a nonrandomized nationwide trial of the KiVa antibullying program for grades 1–9. J Consult Clin Psychol. 2011;79:796–805. 21967491 10.1037/a0025740

[47] Barrett P. Friends for Life - Group leaders’ manual for children. Bowen Hills: Australian Academic Press; 2004.

[48] Sandín B, Chorot P, Valiente RM, Chorpita BF. Development of a 30-item version of the Revised Child Anxiety and Depression Scale. Revista de Psicopatología y Psicología Clínica. 2010;15:165–78.

[49] Chorpita BF, Tracey SA, Brown TA, Collica TJ, Barlow DH. Assessment of worry in children and adolescents: an adaptation of the Penn State Worry Questionnaire. Behav Res Ther. 1997;35:569–81. 9159982 10.1016/s0005-7967(96)00116-7

[50] Rosenberg M. Society and the adolescent self-image. Princeton: Princeton University Press; 2015.

[51] Furber G, Segal L. The validity of the Child Health Utility instrument (CHU9D) as a routine outcome measure for use in child and adolescent mental health services. Health Qual Life Outcomes. 2015;13:22. 25890377 10.1186/s12955-015-0218-4 PMC4340862

[52] Shochet IM, Ham D. Universal school-based approaches to preventing adolescent depression: past findings and future directions of the Resourceful Adolescent Program. Int J Ment Health Promot. 2004;6:17–25.

[53] Angold A, Costello EJ, Messer SC, Pickles A, Winder F, Silver D. The development of a short questionnaire for use in epidemiological studies of depression in children and adolescents. Int J Methods Psychiatr Res. 1995;5:237–49.

[54] Schniering CA, Rapee RM. Development and validation of a measure of children’s automatic thoughts: the children’s automatic thoughts scale. Behav Res Ther. 2002;40:1091–109. 12296494 10.1016/s0005-7967(02)00022-0

[55] Goodenow C. The psychological sense of school membership among adolescents: Scale development and educational correlates. Psychol Sch. 1993;30:79–90.

[56] Radloff LS. The CES-D scale: a self-report depression scale for research in the general population. Appl Psychol Meas. 1977;1:385–401.

[57] Tennant R, Hiller L, Fishwick R, Platt S, Joseph S, Weich S, et al. The Warwick-Edinburgh Mental Well-being Scale (WEMWBS): development and UK validation. Health Qual Life Outcomes. 2007;5:63. 18042300 10.1186/1477-7525-5-63 PMC2222612

[58] Gioia GA, Isquith PK, Guy SC, Kenworthy L. BRIEF-2: Behavior rating inventory of executive function. Florida: Psychological Assessment Resources Lutz; 2015.

[59] Chorpita BF, Yim L, Moffitt CE, Umemoto LA, Francis SE. Assessment of symptoms of DSMIV anxiety and depression in children: A Revised Child Anxiety And Depression Scale. Behav Res Ther. 2000;38:835–55. 10937431 10.1016/s0005-7967(99)00130-8

[60] Spier E. Alaska School Climate and Connectedness Survey: 2016 Statewide Report. California: American Institutes for Research; 2016.

[61] Greco LA, Baer RA, Smith GT. Assessing mindfulness in children and adolescents: development and validation of the Child and Adolescent Mindfulness Measure (CAMM). Psychol Assess. 2011;23:606–14. 21480722 10.1037/a0022819

[62] StataCorp. Stata Statistical Software: Release 18. College Station, TX: StataCorp LLC; 2023.

[63] Gulliford MC, Ukoumunne OC, Chinn S. Components of variance and intraclass correlations for the design of community-based surveys and intervention studies: data from the Health Survey for England 1994. Am J Epidemiol. 1999;149:876–83. 10221325 10.1093/oxfordjournals.aje.a009904

[64] Siddiqui O, Hedeker D, Flay BR, Hu FB. Intraclass correlation estimates in a school-based smoking prevention study: outcome and mediating variables, by sex and ethnicity. Am J Epidemiol. 1996;144:425–33. 8712201 10.1093/oxfordjournals.aje.a008945

[65] Collishaw S, Goodman R, Ford T, Rabe-Hesketh S, Pickles A. How far are associations between child, family and community factors and child psychopathology informant-specific and informant-general? J Child Psychol Psychiatry. 2009;50:571–80. 19207620 10.1111/j.1469-7610.2008.02026.x

[66] Stone LL, Otten R, Engels RC, Vermulst AA, Janssens JM. Psychometric properties of the parent and teacher versions of the strengths and difficulties questionnaire for 4- to 12-year-olds: a review. Clin Child Fam Psychol Rev. 2010;13:254–74. 20589428 10.1007/s10567-010-0071-2 PMC2919684

[67] van den Heuvel M, Jansen D, Stewart RE, Smits-Engelsman BCM, Reijneveld SA, Flapper BCT. How reliable and valid is the teacher version of the Strengths and Difficulties Questionnaire in primary school children? PLoS ONE. 2017;12:e0176605. 28453573 10.1371/journal.pone.0176605 PMC5409073

[68] Department for Education. Behaviour in Schools: Advice for headteachers and school staff. In: Education Df, editor. 2022.

[69] Bradshaw CP, Waasdorp TE, Debnam KJ, Johnson SL. Measuring school climate in high schools: a focus on safety, engagement, and the environment. J Sch Health. 2014;84:593–604. 25117894 10.1111/josh.12186

[70] Kidger J, Araya R, Donovan J, Gunnell D. The effect of the school environment on the emotional health of adolescents: a systematic review. Pediatrics. 2012;129:925–49. 22473374 10.1542/peds.2011-2248

[71] Bonell C, Parry W, Wells H, Jamal F, Fletcher A, Harden A, et al. The effects of the school environment on student health: a systematic review of multi-level studies. Health Place. 2013;21:180–91. 23501377 10.1016/j.healthplace.2012.12.001

[72] Diedrichs PC, Atkinson MJ, Steer RJ, Garbett KM, Rumsey N, Halliwell E. Effectiveness of a brief school-based body image intervention ‘Dove Confident Me: Single Session’when delivered by teachers and researchers: Results from a cluster randomised controlled trial. Behav Res Ther. 2015;74:94–104. 26469131 10.1016/j.brat.2015.09.004

