# Supplementary material for: Patterns of intra-cluster correlation coefficients in school-based cluster randomised controlled trials of interventions for improving social-emotional functioning outcomes in pupils: a secondary data analysis of five UK-based studies
Source: BMC Med Res Methodol. 2025 May 3;25:120. doi: 10.1186/s12874-025-02574-6 (PMC12048950; doi:10.1186/s12874-025-02574-6)
Supplement: Supplementary file 1 — Supplementary Material 1. [file 12874_2025_2574_MOESM1_ESM.docx]

**Supplementary Table S1.** Description of outcomes and measures

| **Author, year (Study acronym)** | **Outcome** | **Outcome measure** | **Type of outcome** | **Number of items, scoring and scoring range** | **Outcome reporter(s)** |
| --- | --- | --- | --- | --- | --- |
| Ford, 2019 [11] (STARS) | Social and emotional functioning | Strengths and Difficulty Questionnaire (SDQ) [41] (Total difficulties score, *emotional symptoms*, *conduct problems*, *hyperactivity*, *peer problem* and *prosocial behaviour* subscales) | Continuous | 25 ordinal items  Each item scored from 0 to 2  20 items are summed to calculate the *total difficulties* score (excluding the 5 items in the *prosocial behaviour subscale*)  5 items are summed to calculate total score for each of 5 subscales  Scoring range for the *total difficulties* score is 0 to 40  Scoring range for each of the 5 subscales is 0 to 10 | Teacher  Parent |
|  | School climate | ‘How I Feel About My School’ (HIFAMS) [43] | Continuous | 7 ordinal items  Each item scored from 0 to 2   - Total score ranges from 0 to 14 | Pupil |
|  | Pupil behaviour | Pupil Behaviour Questionnaire (PBQ) [42] | Continuous | 6 ordinal items  Each item scored from 0 to 2   - Total score ranges from 0 to 12 | Teacher |
| Axford, 2020 [8] (KiVa) | Bullying victimisation and bullying perpetration | Olweus Bully/Victim Questionnaire [45] (Bullying victimisation and bulling perpetration) | Binary^1^ | Bullying victimisation was measured using the item: “How often have you been bullied at school in the last couple of months?”  Bullying perpetration was measured using the item: “How often have you bullied others at school in the last few months?”  Each item scored from 0 to 4 (“Not at all” (0), “Only once or twice” (1), “2-3 times a month” (2), “About once a week” (3), “Several times per week” (4))   - Each item was dichotomised for analysis so that those scoring 2 to 4 were classified as victims/perpetrators and those scoring 0 or 1 as not victims/not perpetrators. | Pupil |
|  | Bullying victimisation and bullying perpetration | KiVa student online survey [46] | Binary | Told school about being bullied (Yes/No)  Did not tell school about being bullied (Yes/No)  Told home about being bullied (Yes/No) | Pupil |
|  | Social and emotional functioning | Strengths and Difficulty Questionnaire (SDQ) [41] (Total difficulties score, *emotional symptoms*, *conduct problems*, *hyperactivity*, *peer problem* and *prosocial behaviour* subscales) | Continuous | 25 ordinal items  Each item scored from 0 to 2  20 items are summed to calculate the *total difficulties* score (excluding the 5 items in the *prosocial behaviour subscale*)  5 items are summed to calculate total score for each of 5 subscales  Scoring range for the *total difficulties* score is 0 to 40   - Scoring range for each of the 5 subscales is 0 to 10 | Teacher |
| Stallard, 2014 [15] (PACES) | Symptoms of anxiety and low mood | Revised Child Anxiety and Depression Scale (RCADS-30) [48] (Total anxiety scale, *separation anxiety disorder (SAD)*, *social phobia*, *generalised anxiety disorder (GAD)*, *panic disorder*, *obsessive compulsive disorder (OCD)*, and *low mood* (*major depressive disorder*) subscales) | Continuous | 30 ordinal items  Subscales: SAD (5 items); Social Phobia (5 items); GAD (5 items); Panic Disorder (5 items); OCD (5 items); low mood (5 items)  Each item scored from 0 to 3  Total anxiety score is the sum of SAD, Social Phobia, GAD, Panic Disorder and OCD subscales.  Scores for total score range from 0 to 111  Subscales scores range: SAD (0 to 15); Social Phobia (0 to 15); GAD (0 to 15); Panic Disorder (0 to 15); OCD (0 to 15); low mood (0 to 15) | Pupil  Parent (RCADS-30-P) |
|  | Worry | Penn State Worry Questionnaire for Children [49] | Continuous | 14 ordinal items  Each item scored from 0 to 3  Scores range from 0 to 42 | Pupil |
|  | Self-worth and acceptance | Rosenberg Self-Esteem Scale [50] | Continuous | - 10 ordinal items - Each item scored from 0-3 - Scores range from 0 to 30 | Pupil |
|  | Bullying victimisation | Olweus Bully/Victim Questionnaire [45] | Binary | Bullying victimisation was measured using the item: “How often have you been bullied at school in the last couple of months?”  Each item scored from 0 to 4 (“Not at all” (0), “Only once or twice” (1), “2-3 times a month” (2), “About once a week” (3), “Several times per week” (4))   - Dichotomised for analysis (scores greater than or equal to 2) - ‘Bullied more than or equal to 2 -3 times per month’ | Pupil |
|  | Life satisfaction | Child Health Utility instrument (CHU9D) [51] | Continuous | - 9 ordinal items - Each item scored from 1 to 5 - Total scores range from 9 to 45 | Pupil |
|  | Social and emotional functioning | Strengths and Difficulty Questionnaire (SDQ) [41] (Total difficulties score, *emotional symptoms*, *conduct problems*, *hyperactivity*, *peer problem* and *prosocial behaviour* subscales) | Continuous | 25 ordinal items  Each item scored from 0 to 2  20 items are summed to calculate the *total difficulties* score (excluding the 5 items in the *prosocial behaviour subscale*)  5 items are summed to calculate total score for each of 5 subscales  Scoring range for the *total difficulties* score is 0 to 40   - Scoring range for each of the 5 subscales is 0 to 10 | Teacher  Parent |
| Stallard, 2012 [14] (PROMISE) | Symptoms of low mood | Short Mood and Feelings questionnaire [53] | Continuous | 13 ordinal items  Each item scored from 0 to 2  Total scores range from 0 to 26 | Pupil |
|  | Negative thinking | Personal Failure subscale of the Children’s Automatic Thoughts Scale (CATS) [54] | Continuous | 10 ordinal items  Each item scored from 0 to 4  Total scores range from 0 to 40 | Pupil |
|  | Self-worth and acceptance | Rosenberg Self-Esteem Scale [50] | Continuous | - 10 ordinal items - Each item scored from 0 to 3   Total scores range from 0 to 30 | Pupil |
|  | Anxiety | Revised Child Anxiety and Depression Scale (RCADS- 30) [48] (Total anxiety scale, *separation anxiety disorder (SAD)*, *social phobia*, *generalised anxiety disorder (GAD)*, *panic disorder*, *obsessive compulsive disorder (OCD)*, and *low mood* (*major depressive disorder*) subscales) | Continuous | 30 ordinal items  Subscales: SAD (5 items); Social Phobia (5 items); GAD (5 items); Panic Disorder (5 items); OCD (5 items); low mood (5 items).  Each item scored from 0 to 3  Total anxiety score is the sum of SAD, Social Phobia, GAD, Panic Disorder and OCD subscales.  Scores for total score range from 0 to 111  Subscales scores range: SAD (0 to 15); Social Phobia (0 to 15); GAD (0 to 15); Panic Disorder (0 to 15); OCD (0 to 15); low mood (0 to 15) | Pupil |
|  | School connectedness | Psychological Sense of School Membership (PSSM) scale [55] | Continuous | 18 ordinal items  Each item scored from 1 to 5  Total scores range from 18 to 90 | Pupil |
| Kuyken, 2022 [35] (MYRIAD) | Risk for depression | Centre for Epidemiologic Studies for Depression Scale (CES-D) [56] | Continuous | - 20 ordinal items - Each item scored from 0 to 3 - Total scores range from 0 to 60 | Pupil |
|  | Social and emotional functioning | Strengths and Difficulty Questionnaire (SDQ) [41] (Total difficulties score, *emotional symptoms*, *conduct problems*, *hyperactivity*, *peer problem* and *prosocial behaviour* subscales) | Continuous | 25 ordinal items  Each item scored from 0 to 2  20 items are summed to calculate the *total difficulties* score (excluding the 5 items in the *prosocial behaviour subscale*)  5 items are summed to calculate total score for each of 5 subscales  Scoring range for the *total difficulties* score is 0 to 40   - Scoring range for each of the 5 subscales is 0 to 10 | Pupil  Teacher |
|  | Well-being | Warwick-Edinburgh Mental Well-being Scale (WEMWBS) [57] | Continuous | - 14 ordinal items - Each item scored from 1 to 5 - Total scores range from 14 to 70 | Pupil |
|  | Executive function | Behaviour Rating Inventory of Executive Function (BRIEF-2) [58] | Continuous | - 55 ordinal items for pupil - 63 ordinal items for teachers - Each item scored from 1 to 3 - Scores range from 55 to 165 for the pupil version and from 63 to 189 for the teacher version | Pupil  Teacher |
|  | Anxiety | Revised Child Anxiety and Depression Scale (RCADS) [59] (Total anxiety scale, *separation anxiety disorder (SAD)*, *social phobia*, *generalised anxiety disorder (GAD)*, *panic disorder*, *obsessive compulsive disorder (OCD)*, and *low mood* (*major depressive disorder*) subscales) | Continuous | 47 ordinal items  Subscales: SAD (7 items); Social Phobia (9 items); GAD (6 items); Panic Disorder (9 items); OCD (6 items); low mood (10 items).  Items in the low mood subscale were not included in the MYRIAD study so only 37 items were administered.  Each item scored from 0 to 3  Total anxiety score is the sum of the 37 items spanning SAD, Social Phobia, GAD, Panic Disorder and OCD subscales.  Scores for total score range from 0 to 111   - Subscales scores range: SAD (0 to 21); Social Phobia (0 to 27); GAD (0 to 18); Panic Disorder (0 to 27); OCD (0 to 18); low mood (0 to 30) | Pupil |
|  | Self-harm and suicidal ideation | Measures devised for study [35] | Binary | - Self-harm: ‘Have you deliberately harmed yourself?’ Response set: “Yes” or “No” - Suicide ideation: ‘Do you feel like your life is not worth living?’ Response set: “Yes” or “No” | Pupil |
|  | School climate | *School climate subscale* *(School leadership and involvement, respectful climate, peer climate, caring adults)* School Climate and Connectedness Survey (SCCS) [60] | Continuous | 4 sub-sections make up school climate subscale  Each sub-section has 5 ordinal items (20 items in total)  Each item scored from 1 to 5   - Scores for each sub-section range from 5 to 25 - Total scores for the school climate subscale range from 20 to 100 | Pupil |
|  | Mindfulness skills | Child and Adolescent Mindfulness Measure (CAMM) [61] | Continuous | 10 ordinal items  Each item scored from 0 to 4   - Total scores range from 0 to 40 | Pupil |

**Supplementary Table S2.** STARS study intra-cluster correlation coefficients (ICCs) at the school level for the teacher- and parent-reported Strengths and Difficulties Questionnaire (SDQ) and the teacher-reported Pupil Behaviour Questionnaire at different time points

| **Outcome** | **Measurement time (months)**^1^ |  | **Teacher report** | | | |  | **Parent report** | | | |
| --- | --- | --- | --- | --- | --- | --- | --- | --- | --- | --- | --- |
|  |  |  | **N** | $\boldsymbol{\sigma}_{\boldsymbol{s}}^{\boldsymbol{2}}$ | $\boldsymbol{\sigma}_{\boldsymbol{e}}^{\boldsymbol{2}}$ | ${}_{\boldsymbol{s}}$ |  | **N** | $\boldsymbol{\sigma}_{\boldsymbol{s}}^{\boldsymbol{2}}$ | $\boldsymbol{\sigma}_{\boldsymbol{e}}^{\boldsymbol{2}}$ | ${}_{\boldsymbol{s}}$ |
| Total difficulties score (SDQ) | 0 |  | 2074 | 4.118 | 30.181 | 0.12 |  | 1466 | 0.915 | 34.799 | 0.026 |
|  | 9 |  | 2001 | 6.114 | 27.896 | 0.18 |  | 1285 | 1.909 | 39.342 | 0.046 |
|  | 18 |  | 1848 | 7.812 | 35.842 | 0.179 |  | 1225 | 1.238 | 38.425 | 0.031 |
|  | 30 |  | 1756 | 4.894 | 35.502 | 0.121 |  | 1125 | 1.512 | 43.246 | 0.034 |
| Emotional symptoms subscale (SDQ) | 0 |  | 2074 | 0.421 | 3.754 | 0.101 |  | 1467 | 0.098 | 3.864 | 0.025 |
|  | 9 |  | 2001 | 0 .854 | 3.37 | 0.202 |  | 1286 | 0.147 | 4.645 | 0.031 |
|  | 18 |  | 1848 | 0.853 | 3.921 | 0.179 |  | 1227 | 0.071 | 4.828 | 0.014 |
|  | 30 |  | 1756 | 0.393 | 3.952 | 0.09 |  | 1126 | 0.109 | 5.569 | 0.019 |
| Conduct problems subscale (SDQ) | 0 |  | 2074 | 0.144 | 2.19 | 0.062 |  | 1467 | 0.035 | 2.61 | 0.013 |
|  | 9 |  | 2001 | 0.237 | 2.324 | 0.092 |  | 1287 | 0.046 | 2.81 | 0.016 |
|  | 18 |  | 1848 | 0.359 | 2.705 | 0.117 |  | 1228 | 0.074 | 2.436 | 0.03 |
|  | 30 |  | 1756 | 0.291 | 2.505 | 0.104 |  | 1127 | 0.004 | 2.967 | 0.001 |
| Hyperactivity subscale (SDQ) | 0 |  | 2074 | 0.509 | 9.028 | 0.053 |  | 1466 | 0.024 | 6.742 | 0.004 |
|  | 9 |  | 2001 | 0.787 | 7.937 | 0.09 |  | 1287 | 0.088 | 7.002 | 0.012 |
|  | 18 |  | 1848 | 0.826 | 8.302 | 0.091 |  | 1227 | 0.061 | 6.576 | 0.009 |
|  | 30 |  | 1756 | 0.601 | 7.787 | 0.072 |  | 1127 | 0.07 | 6.777 | 0.01 |
| Peer problems subscale (SDQ) | 0 |  | 2074 | 0.391 | 2.18 | 0.152 |  | 1466 | 0.056 | 2.584 | 0.021 |
|  | 9 |  | 2001 | 0.288 | 2.138 | 0.119 |  | 1286 | 0.145 | 2.84 | 0.049 |
|  | 18 |  | 1848 | 0.368 | 2.434 | 0.131 |  | 1227 | 0.081 | 2.912 | 0.027 |
|  | 30 |  | 1756 | 0.271 | 2.498 | 0.098 |  | 1126 | 0.14 | 2.952 | 0.045 |
| Prosocial behaviour subscale (SDQ) | 0 |  | 2074 | 1.404 | 4.6 | 0.234 |  | 1467 | 0 | 2.982 | 0 |
|  | 9 |  | 2001 | 1.32 | 3.946 | 0.251 |  | 1287 | 0 | 2.929 | 0 |
|  | 18 |  | 1848 | 1.135 | 4.42 | 0.204 |  | 1228 | 0.021 | 2.786 | 0.007 |
|  | 30 |  | 1756 | 0.839 | 4.289 | 0.164 |  | 1127 | 0 | 2.888 | 0 |
| Pupil Behaviour Questionnaire (PBQ) | 0 |  | 2074 | 0.373 | 5.472 | 0.064 |  |  |  |  |  |
|  | 9 |  | 2001 | 0.507 | 5.401 | 0.086 |  |  |  |  |  |
|  | 18 |  | 1848 | 0.545 | 6.095 | 0.082 |  |  |  |  |  |
|  | 30 |  | 1760 | 0.499 | 5.688 | 0.081 |  |  |  |  |  |

Analyses at 9, 18, 30 months adjusted for trial arm status

$\sigma_{s}^{2}$ – School-level component of variance

$\sigma_{e}^{2}$ – Pupil-level component of variance

${}_{s}$ – School-level ICC

In the teacher report analyses there were 80 schools at baseline and 9 months; 78 schools at 18 months; and 79 schools at 30 months.

In the parent report analyses there were 80 schools at all waves.

**Supplementary Table S3.** STARS study intra-cluster correlation coefficients (ICCs) at the school level for the pupil-reported ‘How I Feel About My School’ measure at different time points

| **Outcome** | **Reporter** | **Measurement time (months)**^1^ | **N** | $\boldsymbol{\sigma}_{\boldsymbol{s}}^{\boldsymbol{2}}$ | $\boldsymbol{\sigma}_{\boldsymbol{e}}^{\boldsymbol{2}}$ | ${}_{\boldsymbol{s}}$ |
| --- | --- | --- | --- | --- | --- | --- |
| ‘How I Feel About My School’ measure | Pupil | 0 | 2053 | 0.302 | 5.45 | 0.052 |
|  |  | 9 | 1986 | 0.466 | 5.549 | 0.077 |
|  |  | 18 | 1886 | 0.728 | 6.153 | 0.106 |
|  |  | 30 | 1760 | 0.85 | 6.829 | 0.111 |

Analyses at 9, 18, 30 months adjusted for trial arm status

$\sigma_{s}^{2}$ – School-level component of variance

$\sigma_{e}^{2}$ – Pupil-level component of variance

${}_{s}$ – School-level ICC

There were 80 schools at baseline, 9 months and 18 months; and 79 schools at 30 months.

**Supplementary Table S4.** KiVa study intra-cluster correlation coefficients (ICCs) at the school level and the classroom level for teacher-reported Strengths and Difficulties Questionnaire (SDQ) and pupil-reported Olweus Bully/Victim Questionnaire (OBVQ) and bullying (KiVa questionnaire) at different time points

| **Outcome** | **Reporter** | **Measurement time**  **(months)^1^** | **N** | $\boldsymbol{\sigma}_{\boldsymbol{s}}^{\boldsymbol{2}}$ | $\boldsymbol{\sigma}_{\boldsymbol{c}}^{\boldsymbol{2}}$ | $\boldsymbol{\sigma}_{\boldsymbol{e}}^{\boldsymbol{2}}$ | ${}_{\boldsymbol{s}}$ | ${}_{\boldsymbol{c}}$ |
| --- | --- | --- | --- | --- | --- | --- | --- | --- |
| Total difficulties score (SDQ) | Teacher | 0 | 2832 | 1.487 | 5.688 | 33.117 | 0.037 | 0.147 |
|  |  | 12 | 2652 | 3.220 | 4.790 | 35.245 | 0.075 | 0.120 |
| Emotional symptoms subscale (SDQ) | Teacher | 0 | 2832 | 0.144 | 0.655 | 3.549 | 0.033 | 0.156 |
|  |  | 12 | 2652 | 0.403 | 0.411 | 3.574 | 0.092 | 0.103 |
| Conduct problems subscale (SDQ) | Teacher | 0 | 2832 | 0.123 | 0.171 | 2.614 | 0.042 | 0.061 |
|  |  | 12 | 2652 | 0.178 | 0.152 | 2.876 | 0.055 | 0.050 |
| Hyperactivity subscale (SDQ) | Teacher | 0 | 2832 | 0.045 | 0.728 | 7.736 | 0.005 | 0.086 |
|  |  | 12 | 2652 | 0.252 | 0.715 | 7.431 | 0.030 | 0.088 |
| Peer problems subscale (SDQ) | Teacher | 0 | 2832 | 0.073 | 0.287 | 2.506 | 0.025 | 0.103 |
|  |  | 12 | 2652 | 0.115 | 0.217 | 2.489 | 0.041 | 0.080 |
| Prosocial behaviour subscale (SDQ) | Teacher | 0 | 2832 | 0.057 | 1.123 | 4.330 | 0.010 | 0.206 |
|  |  | 12 | 2652 | 0.085 | 0.718 | 4.148 | 0.017 | 0.148 |
| Bullying victimisation (OBVQ) | Pupil | 0 | 2876 | 0.002 | 0.006 | 0.183 | 0.012 | 0.034 |
|  |  | 12 | 2581 | 0.003 | 0.005 | 0.134 | 0.019 | 0.036 |
| Bullying perpetration (OBVQ) | Pupil | 0 | 2876 | 0.001 | 0.002 | 0.076 | 0.010 | 0.031 |
|  |  | 12 | 2581 | <0.001 | <0.001 | 0.055 | 0.009 | <0.001 |
| Told school about being bullied  (KiVa questionnaire) | Pupil | 0 | 2876 | 0.001 | 0.002 | 0.108 | 0.013 | 0.019 |
|  |  | 12 | 2581 | 0.001 | 0.002 | 0.073 | 0.009 | 0.032 |
| Did not tell school about being bullied (KiVa questionnaire) | Pupil | 0 | 2876 | 0.002 | 0.006 | 0.161 | 0.010 | 0.036 |
|  |  | 12 | 2581 | 0.002 | 0.003 | 0.115 | 0.018 | 0.029 |
| Told home about being bullied  (KiVa questionnaire) | Pupil | 0 | 2876 | 0.001 | 0.004 | 0.133 | 0.006 | 0.032 |
|  |  | 12 | 2581 | 0.002 | 0.002 | 0.096 | 0.017 | 0.024 |

Analyses at 12 months adjusted for trial arm status

$\sigma_{s}^{2}$ – School-level component of variance

$\sigma_{c}^{2}$ – Class-level component of variance

$\sigma_{e}^{2}$ – Pupil-level component of variance

${}_{s}$ – School-level ICC

${}_{c}$ – Class-level ICC

There were 21 schools at baseline and 20 schools at 12 months.

In the teacher report analyses there were 125 classrooms at baseline and 135 classrooms at 12 months.

In the pupil report analyses there were 137 classrooms at baseline and 135 classrooms at 12 months.

**Supplementary Table S5.** PACES study intra-cluster correlation coefficients (ICCs) at the school level and the classroom level for the parent- and pupil-reported Revised Child Anxiety and Depression Scale (RCADS-30) at different time points

| **Outcome** | **Measurement time (months)**^1^ |  |  |  | | **Parent report** | | |  |  | **Pupil report** | | | |  |
| --- | --- | --- | --- | --- | --- | --- | --- | --- | --- | --- | --- | --- | --- | --- | --- |
|  |  |  | **N** | $\boldsymbol{\sigma}_{\boldsymbol{s}}^{\boldsymbol{2}}$ | $\boldsymbol{\sigma}_{\boldsymbol{c}}^{\boldsymbol{2}}$ | $\boldsymbol{\sigma}_{\boldsymbol{e}}^{\boldsymbol{2}}$ | ${}_{\boldsymbol{s}}$ | ${}_{\boldsymbol{c}}$ |  | **N** | $\boldsymbol{\sigma}_{\boldsymbol{s}}^{\boldsymbol{2}}$ | $\boldsymbol{\sigma}_{\boldsymbol{c}}^{\boldsymbol{2}}$ | $\boldsymbol{\sigma}_{\boldsymbol{e}}^{\boldsymbol{2}}$ | ${}_{\boldsymbol{s}}$ | ${}_{\boldsymbol{c}}$ |
| Total anxiety score | 0 |  | 482 | 0 | 0 | 79.054 | 0 | 0 |  | 1281 | 0 | 9.662 | 227.314 | 0 | 0.041 |
|  | 6 |  | 426 | 0 | 0 | 71.077 | 0 | 0 |  | 1274 | 0.544 | 9.336 | 228.518 | 0.002 | 0.039 |
|  | 12 |  | 406 | 0.986 | 0.458 | 61.231 | 0.016 | 0.007 |  | 1203 | 2.417 | 7.032 | 223.379 | 0.01 | 0.031 |
| Low mood subscale | 0 |  | 560 | 0.042 | 0 | 2.401 | 0.017 | 0 |  | 1332 | 0.052 | 0.094 | 6.461 | 0.008 | 0.014 |
|  | 6 |  | 477 | 0 | 0.018 | 2.472 | 0 | 0.007 |  | 1305 | 0 | 0.188 | 6.414 | 0 | 0.028 |
|  | 12 |  | 445 | 0 | 0.024 | 2.28 | 0 | 0.01 |  | 1250 | 0.089 | 0.191 | 6.41 | 0.013 | 0.029 |
| Separation Anxiety Disorder subscale | 0 |  | 519 | 0 | 0.029 | 5.99 | 0 | 0.005 |  | 1330 | 0.106 | 0.292 | 10.577 | 0.01 | 0.027 |
|  | 6 |  | 448 | 0.103 | 0 | 4.778 | 0.021 | 0 |  | 1308 | 0.217 | 0.354 | 8.946 | 0.023 | 0.038 |
|  | 12 |  | 432 | 0.068 | 0.189 | 4.077 | 0.016 | 0.044 |  | 1247 | 0.235 | 0.263 | 8.582 | 0.026 | 0.03 |
| Social phobia subscale | 0 |  | 558 | 0.055 | 0 | 7.502 | 0.007 | 0 |  | 1328 | 0 | 0.346 | 10.271 | 0 | 0.033 |
|  | 6 |  | 479 | 0 | 0.033 | 7.111 | 0 | 0.005 |  | 1307 | 0.151 | 0.248 | 10.988 | 0.014 | 0.022 |
|  | 12 |  | 441 | 0.15 | 0 | 6.303 | 0.023 | 0 |  | 1244 | 0.071 | 0.298 | 11.018 | 0.006 | 0.026 |
| Generalised Anxiety Disorder subscale | 0 |  | 557 | 0 | 0 | 5.836 | 0 | 0 |  | 1328 | 0 | 0.496 | 13.593 | 0 | 0.035 |
|  | 6 |  | 477 | 0.052 | 0 | 4.47 | 0.011 | 0 |  | 1305 | 0 | 0.521 | 12.956 | 0 | 0.039 |
|  | 12 |  | 444 | 0.092 | 0 | 4.159 | 0.022 | 0 |  | 1242 | 0.022 | 0.489 | 12.71 | 0.002 | 0.037 |
| Panic disorder subscale | 0 |  | 550 | 0.005 | 0 | 1.377 | 0.004 | 0 |  | 1326 | 0 | 0.073 | 8.435 | 0 | 0.009 |
|  | 6 |  | 473 | 0 | 0 | 1.337 | 0 | 0 |  | 1305 | 0 | 0.164 | 8.568 | 0 | 0.019 |
|  | 12 |  | 443 | 0.007 | 0 | 0.898 | 0.007 | 0 |  | 1247 | 0.049 | 0.064 | 7.485 | 0.006 | 0.008 |
| Obsessive-compulsive Disorder subscale | 0 |  | 559 | 0 | 0 | 2.095 | 0 | 0 |  | 1325 | 0 | 0.416 | 9.945 | 0 | 0.04 |
|  | 6 |  | 478 | 0.008 | 0 | 2.098 | 0.004 | 0 |  | 1307 | 0 | 0.279 | 10.315 | 0 | 0.026 |
|  | 12 |  | 444 | 0.011 | 0 | 1.891 | 0.006 | 0 |  | 1245 | 0 | 0.223 | 9.805 | 0 | 0.022 |

Analyses at 6 and 12 months adjusted for trial arm status

$\sigma_{s}^{2}$ – School-level component of variance

$\sigma_{c}^{2}$ – Class-level component of variance

$\sigma_{e}^{2}$ – Pupil-level component of variance

${}_{s}$ – School-level ICC

${}_{c}$ – Class-level ICC

In the parent report analyses there were 40 schools at all study waves; 69 classrooms at baseline, 70 classrooms at 6 months and 69 classrooms at 12 months.

In the pupil report analyses there were 40 schools and 73 classrooms at all study waves.

**Supplementary Table S6.** PACES study intra-cluster correlation coefficients (ICCs) at the school level and the classroom level for the parent-reported Strengths and Difficulties Questionnaire (SDQ) at different time points

| **Outcome** | **Measurement time (months)**^1^ |  | **N** | $\boldsymbol{\sigma}_{\boldsymbol{s}}^{\boldsymbol{2}}$ | $\boldsymbol{\sigma}_{\boldsymbol{c}}^{\boldsymbol{2}}$ | $\boldsymbol{\sigma}_{\boldsymbol{e}}^{\boldsymbol{2}}$ | ${}_{\boldsymbol{s}}$ | ${}_{\boldsymbol{c}}$ |
| --- | --- | --- | --- | --- | --- | --- | --- | --- |
| Total difficulties score | 0 | | 547 | 0.09 | 0.138 | 39.269 | 0.002 | 0.003 |
|  | 6 | | 460 | 0 | 1.248 | 34.869 | 0 | 0.035 |
|  | 12 | | 425 | 1.743 | 0 | 32.8 | 0.05 | 0 |
| Emotional symptoms subscale | 0 | | 566 | 0 | 0 | 5.511 | 0 | 0 |
|  | 6 | | 475 | 0 | 0.001 | 4.213 | 0 | <0.001 |
|  | 12 | | 439 | 0.119 | 0 | 3.663 | 0.032 | 0 |
| Conduct problems subscale | 0 | | 563 | 0 | 0.139 | 2.949 | 0 | 0.045 |
|  | 6 | | 473 | 0 | 0.06 | 2.522 | 0 | 0.023 |
|  | 12 | | 441 | 0.015 | 0 | 2.33 | 0.006 | 0 |
| Hyperactivity subscale | 0 | | 566 | 0 | 0 | 6.42 | 0 | 0 |
|  | 6 | | 475 | 0 | 0.153 | 5.26 | 0 | 0.028 |
|  | 12 | | 437 | 0.051 | 0 | 4.856 | 0.01 | 0 |
| Peer problems subscale | 0 | | 561 | 0.093 | 0.028 | 3.189 | 0.028 | 0.009 |
|  | 6 | | 475 | 0.212 | 0 | 3.459 | 0.059 | 0 |
|  | 12 | | 438 | 0.058 | 0.039 | 3.28 | 0.017 | 0.012 |
| Prosocial behaviour subscale | 0 | | 561 | 0 | 0 | 3.339 | 0 | 0 |
|  | 6 | | 471 | 0 | 0 | 3.296 | 0 | 0 |
|  | 12 | | 440 | 0 | 0 | 2.794 | 0 | 0 |

Analyses at 6 and 12 months adjusted for trial arm status

$\sigma_{s}^{2}$ – School-level component of variance

$\sigma_{c}^{2}$ – Class-level component of variance

$\sigma_{e}^{2}$ – Pupil-level component of variance

${}_{s}$ – School-level ICC

${}_{c}$ – Class-level ICC

There were 40 schools at all study waves; 69 classrooms at baseline, 70 classrooms at 6 months and 69 classrooms at 12 months.

**Supplementary Table S7.** PACES study intra-cluster correlation coefficients (ICCs) at the school level and the classroom level for pupil-reported bully victimisation, worry, self-esteem and life satisfaction at different time points

| **Outcome** | **Measurement time (months)**^1^ | **N** | $\boldsymbol{\sigma}_{\boldsymbol{s}}^{\boldsymbol{2}}$ | $\boldsymbol{\sigma}_{\boldsymbol{c}}^{\boldsymbol{2}}$ | $\boldsymbol{\sigma}_{\boldsymbol{e}}^{\boldsymbol{2}}$ | ${}_{\boldsymbol{s}}$ | ${}_{\boldsymbol{c}}$ |
| --- | --- | --- | --- | --- | --- | --- | --- |
| Bullying victimisation  (Olweus Bully/Victim Questionnaire) | 0 | 1338 | 0.003 | 0.004 | 0.196 | 0.015 | 0.018 |
|  | 6 | 1316 | 0.006 | 0.002 | 0.187 | 0.031 | 0.011 |
|  | 12 | 1254 | 0.001 | 0.008 | 0.154 | 0.005 | 0.051 |
| Worry  (Penn Worry Scale) | 0 | 1310 | 0 | 0.360 | 67.391 | 0 | 0.005 |
|  | 6 | 1298 | 0 | 1.000 | 67.009 | 0 | 0.015 |
|  | 12 | 1230 | 0.694 | 1.314 | 65.922 | 0.010 | 0.020 |
| Self-esteem  (Rosenberg Self-Esteem Scale) | 0 | 1295 | 0 | 1.334 | 29.467 | 0 | 0.043 |
|  | 6 | 1285 | 0.834 | 0.489 | 34.333 | 0.023 | 0.014 |
|  | 12 | 1224 | 0.431 | 1.645 | 34.080 | 0.012 | 0.046 |
| Total life satisfaction  (CHU9D) | 0 | 1333 | 0.328 | 0.821 | 37.111 | 0.009 | 0.022 |
|  | 6 | 1302 | 0.135 | 1.443 | 42.155 | 0.003 | 0.033 |
|  | 12 | 1241 | 1.114 | 1.108 | 38.341 | 0.027 | 0.028 |

Analyses at 6 and 12 months adjusted for trial arm status

$\sigma_{s}^{2}$ – School-level component of variance

$\sigma_{c}^{2}$ – Class-level component of variance

$\sigma_{e}^{2}$ – Pupil-level component of variance

${}_{s}$ – School-level ICC

${}_{c}$ – Class-level ICC

There were 40 schools and 73 classrooms at all study waves.

**Supplementary Table S8.** PROMISE study intra-cluster correlation coefficients (ICCs) at the school level, the year group level and the classroom level for pupil-reported Revised Child Anxiety and Depression Scale (RCADS-30), self-esteem, personal failure, school connectedness, and the Short Moods and Feelings Questionnaire (SMFQ) at different time points

| **Outcome** | **Measurement time (months)**^1^ | **N** | $\boldsymbol{\sigma}_{\boldsymbol{s}}^{\boldsymbol{2}}$ | $\boldsymbol{\sigma}_{\boldsymbol{g}}^{\boldsymbol{2}}$ | $\boldsymbol{\sigma}_{\boldsymbol{c}}^{\boldsymbol{2}}$ | $\boldsymbol{\sigma}_{\boldsymbol{e}}^{\boldsymbol{2}}$ | ${}_{\boldsymbol{s}}$ | ${}_{\boldsymbol{g}}$ | ${}_{\boldsymbol{c}}$ |
| --- | --- | --- | --- | --- | --- | --- | --- | --- | --- |
| Total anxiety score (RCADS-30) | 0 | 4588 | 0.760 | 2.350 | 3.467 | 95.071 | 0.007 | 0.023 | 0.035 |
|  | 6 | 4395 | 0 | 3.093 | 2.905 | 105.624 | 0 | 0.028 | 0.027 |
|  | 12 | 3948 | 0.720 | 3.219 | 3.303 | 108.924 | 0.006 | 0.028 | 0.029 |
| Low mood subscale (RCADS-30) | 0 | 4607 | 0.073 | 0.116 | 0.195 | 6.680 | 0.010 | 0.017 | 0.028 |
|  | 9 | 4416 | 0 | 0.151 | 0.155 | 7.284 | 0 | 0.020 | 0.021 |
|  | 12 | 3954 | 0.039 | 0.154 | 0.209 | 7.493 | 0.005 | 0.020 | 0.027 |
| Panic disorder subscale (RCADS-30) | 0 | 4612 | 0.055 | 0.066 | 0.198 | 5.568 | 0.009 | 0.011 | 0.034 |
|  | 6 | 4422 | 0 | 0.082 | 0.093 | 6.727 | 0 | 0.012 | 0.014 |
|  | 12 | 3957 | 0.029 | 0.074 | 0.126 | 6.172 | 0.005 | 0.012 | 0.020 |
| Social phobia subscale (RCADS-30) | 0 | 4612 | 0 | 0.569 | 0.143 | 8.326 | 0 | 0.063 | 0.017 |
|  | 6 | 4420 | 0.015 | 0.657 | 0.219 | 8.675 | 0.002 | 0.069 | 0.025 |
|  | 12 | 3956 | 0.061 | 0.521 | 0.116 | 9.348 | 0.006 | 0.052 | 0.012 |
| Generalised Anxiety Disorder subscale (RCADS-30) | 0 | 4616 | 0 | 0.115 | 0.160 | 6.992 | 0 | 0.016 | 0.022 |
|  | 6 | 4427 | 0.029 | 0.054 | 0.219 | 7.635 | 0.004 | 0.007 | 0.028 |
|  | 12 | 3958 | 0.061 | 0.107 | 0.158 | 7.693 | 0.008 | 0.013 | 0.020 |
| Separation Anxiety Disorder subscale (RCADS-30) | 0 | 4616 | 0.041 | 0.005 | 0.057 | 2.806 | 0.014 | 0.002 | 0.020 |
|  | 6 | 4426 | 0.009 | 0.013 | 0.071 | 3.384 | 0.002 | 0.004 | 0.020 |
|  | 12 | 3958 | 0.023 | 0.032 | 0.075 | 3.332 | 0.007 | 0.009 | 0.022 |
| Self-esteem  (Rosenberg self-esteem scale) | 0 | 4576 | 0.121 | 0.645 | 0.760 | 26.899 | 0.004 | 0.023 | 0.027 |
|  | 6 | 4392 | 0 | 0.533 | 0.452 | 30.528 | 0 | 0.017 | 0.015 |
|  | 12 | 3944 | 0 | 0.488 | 0.353 | 30.946 | 0 | 0.015 | 0.011 |
| Personal failure  (Children’s Automatic Thoughts Scale (CATS) | 0 | 4596 | 0.420 | 0.419 | 1.127 | 46.767 | 0.009 | 0.009 | 0.024 |
|  | 6 | 4401 | 0.015 | 0.448 | 0.647 | 53.109 | <0.001 | 0.008 | 0.012 |
|  | 12 | 3945 | 0.035 | 0.573 | 1.227 | 48.746 | 0.001 | 0.011 | 0.025 |
| School connectedness (Psychological Sense of School Membership scale) | 0 | 4567 | 0.293 | 0.578 | 0.654 | 37.968 | 0.007 | 0.015 | 0.017 |
|  | 6 | 4367 | 0.699 | 0.489 | 0.682 | 41.364 | 0.016 | 0.011 | 0.016 |
|  | 12 | 3913 | 0.709 | 0.531 | 0.807 | 42.220 | 0.016 | 0.012 | 0.019 |
| Short moods and feelings questionnaire (SMFQ) | 0 | 4784 | 0.238 | 0.320 | 0.740 | 22.149 | 0.010 | 0.014 | 0.032 |
|  | 6 | 4480 | 0.021 | 0.566 | 0.523 | 25.374 | 0.001 | 0.021 | 0.020 |
|  | 12 | 4140 | 0.119 | 0.379 | 0.683 | 24.618 | 0.005 | 0.015 | 0.027 |

Analyses at 6 and 12 months adjusted for trial arm status

$\sigma_{s}^{2}$ – School-level component of variance

$\sigma_{g}^{2}$ – Year group-level component of variance

$\sigma_{c}^{2}$ – Class-level component of variance

$\sigma_{e}^{2}$ – Pupil-level component of variance

${}_{s}$ – School-level ICC

${}_{g}$ – Year group-level ICC

${}_{c}$ – Class-level ICC

There were 8 schools, 28 year groups and 225 classrooms at all study waves.

**Supplementary Table S9.** MYRIAD study intra-cluster correlation coefficients (ICCs) at the school level and the classroom level for pupil- and teacher-reported Strengths and Difficulties Questionnaire (SDQ) at different time points

| **Outcome** | **Measurement time (months)**^1^ |  |  |  | | **Pupil report** | | |  |  | **Teacher report** | | | |  |
| --- | --- | --- | --- | --- | --- | --- | --- | --- | --- | --- | --- | --- | --- | --- | --- |
|  |  |  | **N** | $\boldsymbol{\sigma}_{\boldsymbol{s}}^{\boldsymbol{2}}$ | $\boldsymbol{\sigma}_{\boldsymbol{c}}^{\boldsymbol{2}}$ | $\boldsymbol{\sigma}_{\boldsymbol{e}}^{\boldsymbol{2}}$ | ${}_{\boldsymbol{s}}$ | ${}_{\boldsymbol{c}}$ |  | **N** | $\boldsymbol{\sigma}_{\boldsymbol{s}}^{\boldsymbol{2}}$ | $\boldsymbol{\sigma}_{\boldsymbol{c}}^{\boldsymbol{2}}$ | $\boldsymbol{\sigma}_{\boldsymbol{e}}^{\boldsymbol{2}}$ | ${}_{\boldsymbol{s}}$ | ${}_{\boldsymbol{c}}$ |
| Total difficulties score | 0 |  | 8252 | 1.051 | 0.666 | 40.01 | 0.025 | 0.016 |  |  |  |  |  |  |  |
|  | 12 |  | 8042 | 0.941 | 0.492 | 42.545 | 0.021 | 0.011 |  | 5873 | 1.82 | 5.561 | 28.343 | 0.051 | 0.164 |
|  | 19 |  | 7542 | 0.933 | 0.933 | 45.043 | 0.02 | 0.021 |  | 5522 | 1.918 | 6.61 | 26.967 | 0.054 | 0.197 |
|  | 24 |  | 7225 | 0.792 | 0.358 | 45.289 | 0.017 | 0.008 |  | 4477 | 2.523 | 5.195 | 26.077 | 0.075 | 0.166 |
| Emotional symptoms | 0 |  | 8254 | 0.117 | 0.05 | 6.378 | 0.018 | 0.008 |  |  |  |  |  |  |  |
|  | 12 |  | 8042 | 0.156 | 0.003 | 6.937 | 0.022 | <0.001 |  | 5873 | 0.234 | 0.486 | 3.247 | 0.059 | 0.13 |
|  | 19 |  | 7542 | 0.164 | 0.094 | 7.213 | 0.022 | 0.013 |  | 5522 | 0.156 | 0.605 | 2.882 | 0.043 | 0.173 |
|  | 24 |  | 7226 | 0.146 | 0.013 | 7.162 | 0.02 | 0.002 |  | 4477 | 0.181 | 0.523 | 2.818 | 0.051 | 0.157 |
| Conduct difficulties | 0 |  | 8253 | 0.078 | 0.052 | 3.456 | 0.022 | 0.015 |  |  |  |  |  |  |  |
|  | 12 |  | 8042 | 0.061 | 0.029 | 3.543 | 0.017 | 0.008 |  | 5873 | 0.072 | 0.257 | 2.152 | 0.029 | 0.107 |
|  | 19 |  | 7542 | 0.057 | 0.07 | 3.872 | 0.014 | 0.018 |  | 5522 | 0.032 | 0.321 | 2.249 | 0.012 | 0.125 |
|  | 24 |  | 7226 | 0.044 | 0.062 | 3.85 | 0.011 | 0.016 |  | 4477 | 0.071 | 0.173 | 2.073 | 0.031 | 0.077 |
| Hyperactivity | 0 |  | 8253 | 0.128 | 0.044 | 5.795 | 0.021 | 0.008 |  |  |  |  |  |  |  |
|  | 12 |  | 8042 | 0.087 | 0.049 | 6.174 | 0.014 | 0.008 |  | 5873 | 0.163 | 0.725 | 6.105 | 0.023 | 0.106 |
|  | 19 |  | 7542 | 0.1 | 0.092 | 6.395 | 0.015 | 0.014 |  | 5522 | 0.341 | 0.727 | 6.132 | 0.047 | 0.106 |
|  | 24 |  | 7226 | 0.089 | 0.047 | 6.479 | 0.013 | 0.007 |  | 4477 | 0.374 | 0.63 | 5.749 | 0.055 | 0.099 |
| Peer problems | 0 |  | 8253 | 0.05 | 0.026 | 3.304 | 0.015 | 0.008 |  |  |  |  |  |  |  |
|  | 12 |  | 8042 | 0.06 | 0.024 | 3.435 | 0.017 | 0.007 |  | 5873 | 0.087 | 0.375 | 2.614 | 0.028 | 0.125 |
|  | 19 |  | 7542 | 0.047 | 0.046 | 3.618 | 0.013 | 0.013 |  | 5522 | 0.087 | 0.453 | 2.334 | 0.03 | 0.163 |
|  | 24 |  | 7225 | 0.056 | 0.011 | 3.563 | 0.015 | 0.003 |  | 4477 | 0.121 | 0.411 | 2.277 | 0.043 | 0.153 |
| Prosocial behaviour | 0 |  | 8254 | 0.041 | 0.068 | 3.158 | 0.012 | 0.021 |  |  |  |  |  |  |  |
|  | 12 |  | 8042 | 0.067 | 0.045 | 3.341 | 0.019 | 0.013 |  | 5873 | 0.176 | 1.2 | 5.28 | 0.026 | 0.185 |
|  | 19 |  | 7542 | 0.076 | 0.047 | 3.667 | 0.02 | 0.013 |  | 5522 | 0.266 | 1.177 | 5.358 | 0.039 | 0.18 |
|  | 24 |  | 7226 | 0.087 | 0.034 | 3.901 | 0.022 | 0.009 |  | 4477 | 0.619 | 0.933 | 5.147 | 0.092 | 0.154 |

Analyses at 12, 19 and 24 months adjusted for trial arm status

$\sigma_{s}^{2}$ – School-level component of variance

$\sigma_{c}^{2}$ – Class-level component of variance

$\sigma_{e}^{2}$ – Pupil-level component of variance

${}_{s}$ – School-level ICC

${}_{c}$ – Class-level ICC

In the pupil report analyses there were 84 schools and 389 classrooms at all study waves.

In the teacher report analyses there were: 71 schools and 303 classrooms at 12 months; 73 schools and 304 classrooms at 19 months; 65 schools and 259 classrooms at 24 months.

**Supplementary Table S10.** MYRIAD study intra-cluster correlation coefficients (ICCs) at the school level and the classroom level for pupil- and teacher-reported Behaviour Rating Inventory of Executive Function, Second Edition (BRIEF-2) at different time points

| **Outcome** | **Measurement time (months)^1^** |  |  |  | | **Pupil report** | | |  |  | **Teacher report** | | | |  |
| --- | --- | --- | --- | --- | --- | --- | --- | --- | --- | --- | --- | --- | --- | --- | --- |
|  |  |  | **N** | $\boldsymbol{\sigma}_{\boldsymbol{s}}^{\boldsymbol{2}}$ | $\boldsymbol{\sigma}_{\boldsymbol{c}}^{\boldsymbol{2}}$ | $\boldsymbol{\sigma}_{\boldsymbol{e}}^{\boldsymbol{2}}$ | ${}_{\boldsymbol{s}}$ | ${}_{\boldsymbol{c}}$ |  | **N** | $\boldsymbol{\sigma}_{\boldsymbol{s}}^{\boldsymbol{2}}$ | $\boldsymbol{\sigma}_{\boldsymbol{c}}^{\boldsymbol{2}}$ | $\boldsymbol{\sigma}_{\boldsymbol{e}}^{\boldsymbol{2}}$ | ${}_{\boldsymbol{s}}$ | ${}_{\boldsymbol{c}}$ |
| BRIEF-2 | 12 |  | 6639 | 8.827 | 6.651 | 415.75 | 0.02 | 0.016 |  | 5898 | 26.419 | 97.887 | 491.6 | 0.043 | 0.166 |
|  | 19 |  | 6541 | 7.464 | 9.282 | 469.04 | 0.015 | 0.019 |  | 5534 | 61.127 | 107.38 | 456.75 | 0.098 | 0.19 |
|  | 24 |  | 6617 | 10.473 | 3.231 | 493.27 | 0.021 | 0.007 |  | 4479 | 57.848 | 85.448 | 426.16 | 0.102 | 0.167 |

Analyses at 12, 19 and 24 months adjusted for trial arm status

$\sigma_{s}^{2}$ – School-level component of variance

$\sigma_{c}^{2}$ – Class-level component of variance

$\sigma_{e}^{2}$ – Pupil-level component of variance

${}_{s}$ – School-level ICC

${}_{c}$ – Class-level ICC

In the pupil report analyses there were 84 schools and 387 classrooms at all study waves. In the teacher report analyses there were: 71 schools and 303 classrooms at 12 months; 73 schools and 303 classrooms at 19 months; 65 schools and 260 classrooms at 24 months.

**Supplementary Table S11.** MYRIAD study intra-cluster correlation coefficients (ICCs) at the school level and the classroom level for the pupil-reported Revised Child Anxiety and Depression Scale (RCADS) at different time points

| **Outcome** | **Measurement time (months)^1^** | **N** | $\boldsymbol{\sigma}_{\boldsymbol{s}}^{\boldsymbol{2}}$ | $\boldsymbol{\sigma}_{\boldsymbol{c}}^{\boldsymbol{2}}$ | $\boldsymbol{\sigma}_{\boldsymbol{e}}^{\boldsymbol{2}}$ | ${}_{\boldsymbol{s}}$ | ${}_{\boldsymbol{c}}$ |
| --- | --- | --- | --- | --- | --- | --- | --- |
| Total anxiety score | 12 | 7585 | 12.347 | 0 | 387.102 | 0.031 | 0 |
|  | 19 | 7175 | 12.837 | 5.484 | 433.151 | 0.028 | 0.013 |
|  | 24 | 6987 | 13.237 | 1.623 | 449.604 | 0.028 | 0.004 |
| Separation Anxiety Disorder subscale | 12 | 7599 | 0.239 | 0.037 | 11.072 | 0.021 | 0.003 |
|  | 19 | 7184 | 0.233 | 0.105 | 12.119 | 0.019 | 0.009 |
|  | 24 | 6996 | 0.205 | 0 | 12.283 | 0.016 | 0 |
| Generalised Anxiety Disorder subscale | 12 | 7619 | 0.539 | 0.083 | 17.545 | 0.030 | 0.005 |
|  | 19 | 7196 | 0.526 | 0.193 | 18.723 | 0.027 | 0.010 |
|  | 24 | 7002 | 0.501 | 0.179 | 18.758 | 0.026 | 0.009 |
| Panic Disorder subscale | 12 | 7587 | 0.571 | 0 | 29.551 | 0.019 | 0 |
|  | 19 | 7176 | 0.728 | 0.280 | 34.844 | 0.020 | 0.008 |
|  | 24 | 6989 | 0.858 | 0.169 | 35.821 | 0.023 | 0.005 |
| Social Anxiety subscale | 12 | 7603 | 1.504 | 0.240 | 39.661 | 0.036 | 0.006 |
|  | 19 | 7186 | 1.800 | 0.854 | 42.565 | 0.040 | 0.020 |
|  | 24 | 6998 | 1.530 | 0.470 | 44.571 | 0.033 | 0.010 |
| Obsessive Compulsive Disorder subscale | 12 ^2^ | 7606 | 0.210 | - | 12.752 | 0.016 | - |
|  | 19 | 7191 | 0.251 | 0.115 | 13.872 | 0.018 | 0.008 |
|  | 24 | 7001 | 0.246 | 0.017 | 13.993 | 0.017 | 0.001 |

Analyses at 12, 19 and 24 months adjusted for trial arm status

$\sigma_{s}^{2}$ – School-level component of variance

$\sigma_{c}^{2}$ – Class-level component of variance

$\sigma_{e}^{2}$ – Pupil-level component of variance

${}_{s}$ – School-level ICC

${}_{c}$ – Class-level ICC

^2^ Three-level model did not converge. Fitted two-level model allowing for clustering at school level only

There were 84 schools and 388 classrooms at all study waves.

**Supplementary Table S12.** MYRIAD study intra-cluster correlation coefficients (ICCs) at the school level and the classroom level for the pupil-reported Centre for Epidemiologic Studies for Depression Scale, Warwick-Edinburgh Mental Well-being Scale, Child and Adolescent Mindfulness Measure, suicide ideation and self-harm at different time points

| **Outcome** | **Measurement time (months)**^1^ | **N** | $\boldsymbol{\sigma}_{\boldsymbol{s}}^{\boldsymbol{2}}$ | $\boldsymbol{\sigma}_{\boldsymbol{c}}^{\boldsymbol{2}}$ | $\boldsymbol{\sigma}_{\boldsymbol{e}}^{\boldsymbol{2}}$ | ${}_{\boldsymbol{s}}$ | ${}_{\boldsymbol{c}}$ |
| --- | --- | --- | --- | --- | --- | --- | --- |
| Centre for Epidemiologic Studies for Depression Scale (CES-D) | 0 | 8370 | 1.518 | 0.932 | 95.276 | 0.016 | 0.010 |
|  | 12 | 8054 | 2.862 | 1.173 | 118.292 | 0.023 | 0.010 |
|  | 19 | 7561 | 2.570 | 2.662 | 131.886 | 0.019 | 0.020 |
|  | 24 | 7238 | 2.660 | 2.084 | 136.147 | 0.019 | 0.015 |
| Warwick-Edinburgh Mental Well-being Scale (WEMWBS) | 0 | 8333 | 1.454 | 1.917 | 91.341 | 0.015 | 0.021 |
|  | 12 | 8058 | 1.559 | 1.535 | 78.882 | 0.019 | 0.019 |
|  | 19 | 7572 | 1.549 | 1.640 | 86.517 | 0.017 | 0.019 |
|  | 24 | 7244 | 1.541 | 1.364 | 93.362 | 0.016 | 0.014 |
| Child and Adolescent Mindfulness Measure (CAMM) | 12 | 7924 | 1.175 | 0.314 | 60.508 | 0.019 | 0.005 |
|  | 19 | 7472 | 1.626 | 1.064 | 65.942 | 0.024 | 0.016 |
|  | 24 | 7171 | 1.483 | 0.678 | 71.924 | 0.020 | 0.009 |
| Suicide ideation | 12 | 6698 | 0.002 | 0.002 | 0.151 | 0.011 | 0.011 |
|  | 19 | 6497 | 0.002 | 0.002 | 0.170 | 0.013 | 0.013 |
|  | 24 | 6322 | 0.002 | 0.002 | 0.176 | 0.012 | 0.010 |
| Self-harm | 12 | 7232 | <0.001 | <0.001 | 0.075 | 0.006 | 0.005 |
|  | 19 | 6820 | 0.001 | 0.001 | 0.093 | 0.011 | 0.011 |
|  | 24 | 6598 | 0.001 | 0.002 | 0.101 | 0.005 | 0.017 |

Analyses at 12, 19 and 24 months adjusted for trial arm status

$\sigma_{s}^{2}$ – School-level component of variance

$\sigma_{c}^{2}$ – Class-level component of variance

$\sigma_{e}^{2}$ – Pupil-level component of variance

${}_{s}$ – School-level ICC

${}_{c}$ – Class-level ICC

There were 84 schools and 389 classrooms at all study waves.

**Supplementary Table S13.** MYRIAD study intra-cluster correlation coefficients (ICCs) at the school level and the classroom level for the pupil-reported School Climate and Connectedness Survey (SCCS) at different time points

| **Outcome** | **Measurement time (months)**^1^ | **N** | $\boldsymbol{\sigma}_{\boldsymbol{s}}^{\boldsymbol{2}}$ | $\boldsymbol{\sigma}_{\boldsymbol{c}}^{\boldsymbol{2}}$ | $\boldsymbol{\sigma}_{\boldsymbol{e}}^{\boldsymbol{2}}$ | ${}_{\boldsymbol{s}}$ | ${}_{\boldsymbol{c}}$ |
| --- | --- | --- | --- | --- | --- | --- | --- |
| Total score | 12 | 7805 | 0.019 | 0.016 | 0.350 | 0.050 | 0.044 |
|  | 19 | 7332 | 0.019 | 0.013 | 0.384 | 0.046 | 0.032 |
|  | 24 | 7087 | 0.017 | 0.009 | 0.398 | 0.039 | 0.021 |
| School leadership and student involvement subscale | 12 | 7843 | 0.035 | 0.028 | 0.577 | 0.055 | 0.046 |
|  | 19 | 7355 | 0.053 | 0.019 | 0.662 | 0.072 | 0.028 |
|  | 24 | 7117 | 0.050 | 0.022 | 0.676 | 0.067 | 0.031 |
| Respectful climate subscale | 12 | 7838 | 0.033 | 0.022 | 0.533 | 0.057 | 0.040 |
|  | 19 | 7346 | 0.031 | 0.015 | 0.577 | 0.050 | 0.025 |
|  | 24 | 7109 | 0.022 | 0.011 | 0.597 | 0.035 | 0.018 |
| Peer climate subscale | 12 | 7826 | 0.035 | 0.013 | 0.487 | 0.066 | 0.025 |
|  | 19 | 7343 | 0.035 | 0.014 | 0.518 | 0.062 | 0.026 |
|  | 24 | 7104 | 0.031 | 0.007 | 0.513 | 0.057 | 0.014 |
| Caring adults subscale | 12 | 7812 | 0.026 | 0.013 | 0.630 | 0.039 | 0.020 |
|  | 19 | 7337 | 0.028 | 0.017 | 0.704 | 0.037 | 0.024 |
|  | 24 | 7094 | 0.025 | 0.007 | 0.749 | 0.032 | 0.009 |

Analyses at 12, 19 and 24 months adjusted for trial arm status

$\sigma_{s}^{2}$ – School-level component of variance

$\sigma_{c}^{2}$ – Class-level component of variance

$\sigma_{e}^{2}$ – Pupil-level component of variance

${}_{s}$ – School-level ICC

${}_{c}$ – Class-level ICC

There were 84 schools at all study waves; 388 classrooms for *Total Score* and *Caring Adults* subscale at 12 months; 389 classrooms for *School Leadership and Student Involvement*, *Respectful Climate* and *Peer Climate* subscales at 12 months; 388 classrooms for *Total Score* and all subscales at 19 months; 389 classrooms for *Total Score* and all subscales at 24 months.
